# Sporulation in solventogenic and acetogenic clostridia

**DOI:** 10.1007/s00253-021-11289-9

**Published:** 2021-04-26

**Authors:** Mamou Diallo, Servé W. M. Kengen, Ana M. López-Contreras

**Affiliations:** 1 Wageningen Food and Biobased Research, Wageningen, The Netherlands; 2 Laboratory of Microbiology, Wageningen University & Research, Wageningen, The Netherlands

**Keywords:** Solventogenic clostridia, Sporulation, Quorum sensing, Sigma factors, ABE production, Acetogens

## Abstract

The *Clostridium* genus harbors compelling organisms for biotechnological production processes; while acetogenic clostridia can fix C1-compounds to produce acetate and ethanol, solventogenic clostridia can utilize a wide range of carbon sources to produce commercially valuable carboxylic acids, alcohols, and ketones by fermentation. Despite their potential, the conversion by these bacteria of carbohydrates or C1 compounds to alcohols is not cost-effective enough to result in economically viable processes. Engineering solventogenic clostridia by impairing sporulation is one of the investigated approaches to improve solvent productivity. Sporulation is a cell differentiation process triggered in bacteria in response to exposure to environmental stressors. The generated spores are metabolically inactive but resistant to harsh conditions (UV, chemicals, heat, oxygen). In *Firmicutes*, sporulation has been mainly studied in bacilli and pathogenic clostridia, and our knowledge of sporulation in solvent-producing or acetogenic clostridia is limited. Still, sporulation is an integral part of the cellular physiology of clostridia; thus, understanding the regulation of sporulation and its connection to solvent production may give clues to improve the performance of solventogenic clostridia. This review aims to provide an overview of the triggers, characteristics, and regulatory mechanism of sporulation in solventogenic clostridia. Those are further compared to the current knowledge on sporulation in the industrially relevant acetogenic clostridia. Finally, the potential applications of spores for process improvement are discussed.

Key Points

*• The regulatory network governing sporulation initiation varies in solventogenic clostridia.*

*• Media composition and cell density are the main triggers of sporulation.*

*• Spores can be used to improve the fermentation process.*

## Introduction

As a growing part of the world’s population is getting access to affordable energy, the global energy demand is increasing drastically. The energy consumed mainly originates from fossil resources, resulting in an acceleration of the depletion of natural resources and increased greenhouse gas (GHG) emissions. To inverse this trend, our societies are transitioning towards more sustainable economies, and countries worldwide are promoting the renewable energy sector. While substitutions to fossil energy generation processes such as hydrothermal, geothermal, solar, or wind energy are promoted, few alternatives to oil for freight, aviation, or the petrochemical sector are cost-effective. “Advanced biofuels,” defined by the International Energy Agency (IEA) as liquid or gaseous fuels derived from lignocellulosic (second-generation biofuel) or algal biomass (third-generation biofuel), are among the most promising substitutes to oil. These feedstocks have a relatively diverse composition and are available as complex polymeric structures (lignocellulose) that require dedicated enzymes to release the fermentable sugars. Some bacterial species from the *Clostridium* genus can hydrolyze these polymers, and ferment the carbohydrates to produce solvents. Clostridia are anaerobic and spore-forming Gram-positive bacteria, and the entire *Clostridium* genus currently comprises over 270 species (https://www.bacterio.net/), including pathogenic, probiotic, thermophilic, and benign soil bacteria. However, the *Clostridium* genus proposed by Prazmowski in 1880 (Prazmowski 1880) is not a monophyletic group (Collins et al. 1994; Jones 2001; Yutin and Galperin 2013), and only a subset of the 16S rRNA tree (cluster I), *Clostridium sensu stricto* is currently recognized as the genus’s representative (Cruz-Morales et al. 2019; Gupta and Gao 2009; Lawson and Rainey 2016). Several non-pathogenic clostridia have been studied for the production of advanced fuels and other biochemicals (Tracy et al. 2012). These species are commonly divided into acid-producing, solvent-producing, cellulolytic, and acetogenic species (Dürre 2005). These bacteria convert simple and complex carbon sources, from C1 compounds to cellulose, into a diverse range of metabolites, ranging from carboxylic acids such as acetate or butyrate to solvents like butanol and propanol. Ten *Clostridium* species are known to be solventogenic (Poehlein et al. 2017), see Table 1, with *C. acetobutylicum, C. beijerinckii, C. pasteurianum, C. saccharobutylicum*, and *C. saccharoperbutylacetonicum* being the most studied. These species have been used industrially during the twentieth century for acetone production through the ABE fermentation process (Berezina et al. 2012; Jones and Woods 1986; Jones 2001; Sauer 2016), but other solventogenic strains with high solvent productivity were isolated recently (Xin et al. 2018).

**Table 1:** Solventogenic clostridia available in international strain collections and main fermentation products

Specie	Products	Reference
*C. acetobutylicum*	Butanol, ethanol, acetone, acetate, butyrate, propanediol	(Forsberg 1987; Jones and Woods 1986; Keis et al. 2001; Shaheen et al. 2000)
*C. beijerinckii*	Butanol, ethanol, acetone, acetate, butyrate, 2,3-butanediol, 2- propanol, propionate, n-propanol, propanediol, isopropanol	(Diallo et al. 2018; Forsberg 1987; Keis et al. 2001; Mate de Gerando et al. 2018; Raedts et al. 2014; Sedlar et al. 2021; Shaheen et al. 2000)
*C. pasteurianum*	Butanol, ethanol, acetone, acetate, butyrate	(Gallazzi et al. 2015; Malaviya et al. 2012; Xin et al. 2016)
*C. saccharobutylicum*	Butanol, ethanol, acetone, acetate, butyrate	(Jones and Woods 1986; Keis et al. 2001; Shaheen et al. 2000)
*C. saccharoper-butylacetonicum*	Butanol, ethanol, acetone, acetate, butyrate	(Jones and Woods 1986; Keis et al. 2001; Shaheen et al. 2000)
*C. puniceum*	Butanol, acetone, acetate, butyrate	(Berezina et al. 2012; Lund et al. 1981)
*C. aurantibutyricum*	Butanol, ethanol, acetone, acetate, butyrate, isopropanol	(George et al. 1983; Jones and Woods 1986)
*C. felsineum*	Butanol, ethanol, acetone, acetate, butyrate	(Avrova et al. 1981; Poehlein et al. 2017; Sjolander et al. 1938)
*C. tetanomorphum*	Butanol, ethanol, acetate, butyrate	(Gong et al. 2016; Patakova et al. 2014)
*C. roseum*	Butanol, ethanol, acetone, acetate, butyrate	(Abd-Alla et al. 2017; Poehlein et al. 2017)

Despite many efforts, bioprocesses relying on these bacteria are not cost-effective due to high feedstock costs, expensive pretreatment of the feedstock, poor substrate use, and low solvent productivity (Green 2011; Tashiro et al. 2013). These issues need to be tackled to enable competitive biofuel prices. According to estimates, the product titer in a bioprocess aiming for the biofuel market needs to reach at least 50 g L^-1,^ and the productivity should amount to 3 g L^-1^ h^-1^ to be commercially viable (Vees et al. 2020). While in standard batch conditions, solvent productivity has been reported to reach 10 g L^-1^ h^-1^ with clostridial fermentation, the clostridial maximum butanol titer in batch fermentation could not exceed 25 g L^-1^ without the application of in situ solvent removal techniques to prevent toxicity (Annous and Blaschek 1991; Qureshi and Blaschek 2001; Wang et al. 2019; Xu et al. 2015). It appears that both solvent toxicity and sporulation prevent the production of higher solvent titers (Cheng et al. 2019; Papoutsakis 2008). During the fermentation, bacteria are grown under stringent conditions, which are necessary for solvent production yet harsh for the cells. As a defense mechanism, the bacteria differentiate into highly resistant cells called endospores (henceforth designated as spores) while producing solvents. Spores are metabolically inactive, and their formation not only requires metabolic energy but also impairs solvent productivity (Tracy et al. 2012). Several efforts were made to engineer asporogenous solvent-producing strains, to prevent these undesirable effects (Al-Hinai et al. 2014; Bi et al. 2011; Scotcher and Bennett 2005). The regulation of sporulation and solventogenesis appears to be coupled, but the underlying regulatory networks remain unclear (Patakova et al. 2013). Due to the lack of efficient engineering tools for *Clostridium*, studies on the regulation of sporulation have been scarce. The recent development of markerless tools for clostridia (Atmadjaja et al. 2019; Cañadas et al. 2019; Diallo et al. 2020a; Huang et al. 2019; Joseph et al. 2018; Li et al. 2019; Seys et al. 2020; Wasels et al. 2020; Zhao et al. 2019) has made the genetic engineering of clostridia much more attainable. As a result, the number of engineered clostridia increased substantially and together with the rise of omics studies, the current knowledge on sporulation in solventogenic clostridia has expanded considerably.

## Sporulation regulation in solventogenic clostridia

In response to changes in the environment, some bacteria produce spores to survive under unfavorable conditions. Depending on the formation mechanism and the structure, different spore types can be found in the environment (Paul et al. 2019). The spores, formed by *Firmicutes*, called endospores (Dürre 2014; Johnson 2019), are the most resilient. Endospores can survive harsh treatments such as high temperatures, the presence of oxygen (for anaerobic bacteria), desiccation, lysozyme incubation, ionizing radiation, and chemical solvents. The most studied sporulating bacteria belong to the *Bacillus* and *Clostridium* genera*.* The sporulation process was first described in *Bacillus* (Dawes and Mandelstam 1970; Kay and Warren 1968; Knaysi 1948; Tokuyasu and Yamada 1959)*,* the model organism among spore formers, and the main features of its sporulation process are conserved in the *Clostridium* genus. Still, substantial differences in the spore morphology and sporulation initiation have been demonstrated between the two genera and within the *Clostridium* genus (Al-Hinai et al. 2015; Dürre 2014).

**Fig. 1:**
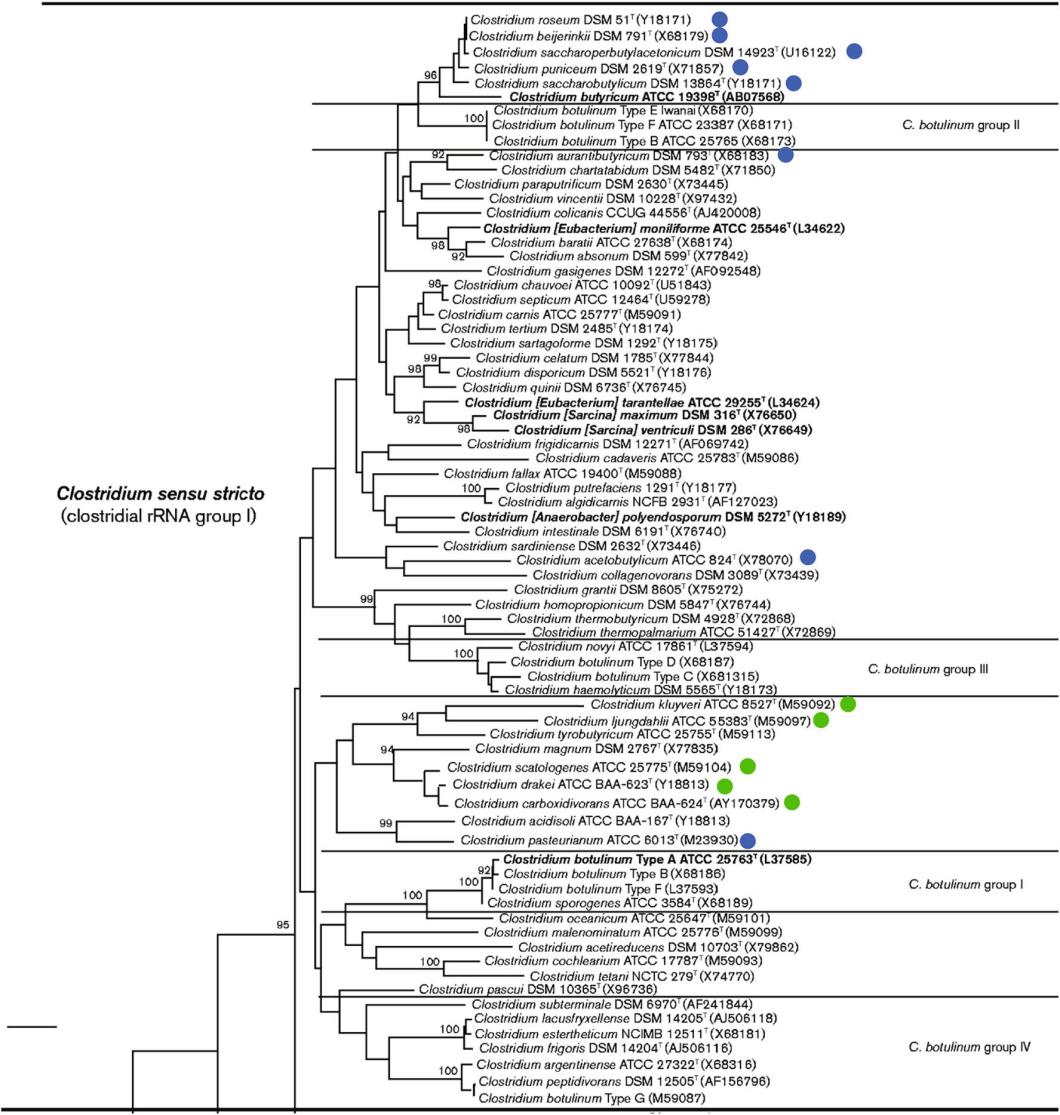
Phylogenetic tree of the *Clostridium sensu stricto* group, amended from (Lawson and Rainey 2016) with permission of the Microbiology Society. The tree was reconstructed using the neighbor-joining method based on the pairwise comparison of approximately 1340  nt. *Atopobium parvulum* was used as the outgroup. Bootstrap values (> 90 %), expressed as a percentage of 1000 replications. Bar, 1 % sequence divergence. A blue disc next to the name of a The strains next to in the blue discs and purple boxes belong indicates that this strain belongs to the solventogenic group, while a green disk next to the name of a strain indicates that this strain belongs to the and the acetogenic clostridia

A sporulation model for the solventogenic clostridia (Al-Hinai et al. 2015) has been developed thanks to studies in *C. acetobutylicum* ATCC 824. Few studies were done on sporulation in other solventogenic clostridia strains to confirm the universality of this model. Although solventogenic clostridia are often presented as a homogenous group of bacteria, based on the first phylogenic studies on the *Clostridium* genus (Collins et al. 1994; Keis et al. 1995), this is not the case. Several strains were renamed and reclassified since 2000 (Keis et al. 2001), and recent phylogenic studies (Cruz-Morales et al. 2019; Yu et al. 2019) show that *C. beijerinckii* and *C. acetobutylicum* even belong to two different clades. Out of the seventeen clades dividing the *Clostridium* genus “sensu stricto,” solventogenic clostridia can be found in two groups, one harboring *C. acetobutylicum* and *C. pasteurianum* and another consisting of *C. beijerinckii*, *C. saccharoperbutylacetonicum*, and *C. saccharobutylicum*. Phylogenetically, *C. beijerinckii* is closer to the human pathogens *C. perfringens* and *C. botulinum* E than to the model solventogenic clostridia *C. acetobutylicum*, as depicted in Fig. 1. In line with what has been suggested for toxin genes (Cruz-Morales et al. 2019), solventogenesis genes might have been acquired by horizontal transfer. The localization of the *sol* operon, harboring the essential solvent genes, on a megaplasmid in *C. acetobutylicum* contrasting with the chromosomal *sol* operon in other clostridia supports this hypothesis. Thus, the regulation mechanisms described for *C. acetobutylicum* might not be identical in *C. beijerinckii* or other solventogenic species (Patakova et al. 2013).

### The sporulation cycle

#### The initiation of sporulation

The sporulation regulation network described in *Bacillus* has been used as a template for understanding the sporulation cascade in *Clostridium* (Davidson et al. 2018; Decker and Ramamurthi 2017; Paredes et al. 2005; Piggot and Hilbert 2004). However, several sporulation genes identified in *Bacillus* are absent in *Clostridium*, indicating a difference in the molecular regulation. Genome comparison studies were conducted to identify homologs to the sporulation genes studied in *B. subtilis* and the necessary set of genes required for sporulation (Galperin 2013; Galperin et al. 2012; Traag et al. 2013). Fifty-two genes were identified as essential for sporulation as they were found both in sporulating clostridia and bacilli by comparing more than 217 genomes of sporulating and non-sporulating *Firmicutes*. Even when gene homologs are found in *Clostridium*, their functions are not always identical to their homologs in *Bacillus* (Al-Hinai et al. 2015; Fimlaid and Shen 2015). Genome comparison, coupled with transcriptomic studies, enabled the identification of additional sporulation genes in solventogenic clostridia (Grimmler et al. 2011; Lee et al. 2015; Máté de Gérando et al. 2018; Sedlar et al. 2018). The role of the central sporulation regulators and sigma factors were studied in solventogenic clostridia through the generation of asporogenous mutants, mainly in *C. acetobutylicum* ATCC 824 and more recently in other solventogenic clostridia (Table 2).

**Table 2:** Mutant strains generated from solventogenic clostridia with changes in sporulation compared to the wild type. When a gene is disrupted or its expression is reduced, “–“ is added; when the gene is overexpressed, ‘+’ is added next to the gene abbreviation. NA stands for “data not available”

	Parent strain	Genotype	Sporulation	ABE production	Method	Reference
*C. acetobutylicum*	ATCC 824	*spo0A*^**-**^	No sporulation	Important decrease	Double-crossover chromosomal integration	(Harris et al. 2002)
	ATCC 824	*spo0A*^**-**^	Reduced sporulation	Important decrease	Allelic coupled exchange (ACE)	(Ehsaan et al. 2016)
	ATCC 824 Δ*spo0A*	*spo0A*^**+**^; native promoter	Early sporulation	NA	Allelic coupled exchange (ACE)	(Ehsaan et al. 2016)
	ATCC 824 Δ*spo0A*	*spo0A*^**+**^; ferredoxin promoter	Early sporulation	NA	Allelic coupled exchange (ACE)	(Ehsaan et al. 2016)
*C. acetobutylicum*	ATCC 824	*spo0A*^**-**^	Reduced sporulation	Important decrease	CRISPR-Cas9i	(Li et al. 2016)
	ATCC 824	*spo0A*^**+**^; native promoter + 43 bp from the upstream *spoIVB*	Early and increased sporulation	Similar to WT	Plasmid overexpression	(Alsaker et al. 2004; Harris et al. 2002)
	ATCC 824	*spo0A*-G179S	Reduced sporulation and no heat resistance	No production	Double-crossover allelic exchange	(Foulquier et al. 2019)
	ATCC 824	*lys*^**-**^	Reduced sporulation	Similar to WT	Clostron	(Liu et al. 2015)
	ATCC 824	*spoIIE* ^**-**^	Reduced sporulation	Increase	antisense RNA	(Scotcher and Bennett 2005)
	ATCC 824	*spoIIE* ^**-**^	No sporulation	Slight decrease (30%)	single-crossover chromosomal integration	(Bi et al. 2011)
	ATCC 824	*sigE* ^**-**^	No sporulation	Similar but inoculum dependent	single-crossover chromosomal integration	(Tracy et al. 2011)
	ATCC 824	*sigF*^**-**^	No sporulation	Similar but inoculum dependent	single-crossover chromosomal integration	(Jones et al. 2011)
	ATCC 824	*sigG* ^**-**^	No sporulation	Similar to WT	single-crossover chromosomal integration	(Tracy et al. 2011)
	ATCC 824	*sigK*^**-**^	No sporulation	Important decrease	double-crossover chromosomal integration	(Al-Hinai et al. 2014)
	ATCC 824 Δ*sigK*	*sigK*^**-**^**,** *spo0A*^**+**^; *ptb* promoter	No sporulation	Similar to WT	Double-crossover chromosomal integration	(Al-Hinai et al. 2014)
*C. acetobutylicum*	ATCC 824	*cac0437* ^**-**^	Increased sporulation	NA	Clostron	(Steiner et al. 2011)
	ATCC 824	*cac0437*^**+**^	Reduced sporulation	NA	Clostron	(Steiner et al. 2011)
	ATCC 824	*cac0323* ^**-**^	Reduced sporulation	NA	Clostron	(Steiner et al. 2011)
	ATCC 824	*cac0903* ^**-**^	Reduced sporulation	NA	Clostron	(Steiner et al. 2011)
	ATCC 824	*cac3319* ^**-**^	Reduced sporulation	NA	Clostron	(Steiner et al. 2011)
	ATCC 824	*cac3319*^**-**^	No sporulation	Increased production	Clostron	(Xu et al. 2017)
	ATCC 824	*cac0323*^***-***^*; cac0903*^***-***^	No sporulation	NA	Clostron	(Steiner et al. 2011)
	ATCC 824	*cac0323*^***--***^*; cac3319*^***-***^	No sporulation	NA	Clostron	(Steiner et al. 2011)
	ATCC 824	*cac3319* ^***-***^*; cac0437* ^**-**^	Increased sporulation	NA	Clostron	(Steiner et al. 2011)
	ATCC 824	*cac0903* ^***-***^*; cac0437* ^**-**^	Increased sporulation	NA	Clostron	(Steiner et al. 2011)
	ATCC 824	*abrB310*^**-**^	Reduced sporulation	Important decrease	Antisense RNA	(Scotcher et al. 2005)
	ATCC 824	*agrA*^**-**^	Reduced sporulation	Similar to WT	Clostron	(Steiner et al. 2012)
	ATCC 824	*agrB*^**-**^	Reduced sporulation	Similar to WT	Clostron	(Steiner et al. 2012)
	ATCC 824	*agrC*^**-**^	Reduced sporulation	Similar to WT	Clostron	(Steiner et al. 2012)
	ATCC 824	*pks*^***-***^	No sporulation	Increased production	Double-crossover allelic exchange	(Herman et al. 2017)
	ATCC 824	*qsrB*^**+**^	Increased sporulation	Important decrease	Plasmid overexpression	(Kotte et al. 2020)
	ATCC 824	*qsrG*^***-***^	Decrease in heat resistant spores	Decreased production (about 30%)	Clostron	(Kotte et al. 2020)
	ATCC 824	*ccpA*^***-***^	Delayed and reduced	Similar to WT with pH control	Targetron	(Ren et al. 2012)
	ATCC 824	*ptb*	No sporulation	Important decrease	antisense RNA	(Desai and Papoutsakis 1999)
	ATCC 824	SNPs in 8 sporulation genes + SNPs in the promoter of 2 sporulation genes	No sporulation	Increased production	Random mutagenesis and directed evolution selection	(Hu et al. 2011)
	ATCC 4259	Unknown	No sporulation	Increased production	Random mutagenesis	(Jain et al. 1994)
	CGMCC 5234	*spo0A*^**-**^	Reduced sporulation	Important decrease	Clostron	(Chen et al. 2013)
*C. beijerinckii*	NCIMB 8052	*spo0A*^***-***^	No sporulation	Important decrease	Chromosomal integration	(Ravagnani et al. 2000; Wilkinson et al. 1995)
	NCIMB 8052	*spo0A*^***-***^	Reduced sporulation	Decreased production (about 15%)	CRISPR-Cas9i	(Li et al. 2016)
	NRRL B-598	*spo0A*^**+**^; ferredoxin promoter	No sporulation	Important decrease	Plasmid overexpression	(Kolek et al. 2017)
	NCIMB 8052	*spoIIE* ^**-**^	No sporulation	Increased production	CRISPR-Cas9	(Diallo et al. 2020a, 2020b)
*C. beijerinckii*	NCIMB 8052	2 SNPs (*cbei_3078* and *cbei_4400*)	Reduced and delayed sporulation	Important decrease	Random mutagenesis and directed evolution selection	(Seo et al. 2021; Shi and Blaschek 2008)
	NCIMB 8052	Unknown (the authors suggest SNPs in *cbei_0769* and *cbei_4400*)	Delayed sporulation	Increased production	Random mutagenesis and directed evolution selection	(Sandoval-Espinola et al. 2013)
	SA-1	2 SNPs (0A box of *abrB* homolog *cbei_4895*)	No sporulation	Important decrease	Random mutagenesis and directed evolution selection	(Seo et al. 2017a)
	NCIMB 8052	Unknown	No sporulation	Important decrease	Random mutagenesis	(Jiao et al. 2016; Zhang et al. 2017)
	CC101	*cbei_2073* ^**-**^	Decrease in heat resistant spores	Increased production	CRISPR-Cas9n	(Xin et al. 2020)
	CC101	*cbei_4484*^**-**^	Decrease in heat resistant spores	Increased production	CRISPR-Cas9n	(Xin et al. 2020)
	NCIMB 8052	*sigL*^**-**^	Decrease in heat resistant spores	No production	CRISPR-Cas9	(Hocq et al. 2019)
	NRRL B-593	*sigL*^**-**^ ; 14 additional SNPs	Decrease in heat resistant spores	No production	Random mutagenesis and directed evolution selection	(Hocq et al. 2019)
*C. pasteurianum*	ATCC 6013	*spo0A*^**-**^; 66 additional SNPs	No sporulation	Increased production	Random mutagenesis and directed evolution selection	(Sandoval et al. 2015)
	ATCC 6013	*spo0A*^***-***^	No sporulation	Increased production	Double-crossover allelic exchange	(Sandoval et al. 2015)
	DSM 525-H1	*spo0A*^***-***^	No sporulation	Increased production	Allelic coupled exchange (ACE)	(Schwarz et al. 2017)
*C. saccharo-butylicum*	NCP 262	*spo0A*-G172S	Reduced sporulation no heat resistance	No production	Double-crossover allelic exchange	(Foulquier et al. 2019)
*C. saccharoperbutyl-acetonicum*	N1-4(HMT)	*spo0A*-I261T	No sporulation	Similar to WT	Endogenous CRISPR-Cas	(Atmadjaja et al. 2019)
	N1-4(HMT)	*qss1*^**-**^	Increased sporulation	Similar to WT with pH control	CRISPR-Cas9	(Feng et al. 2020)
	N1-4(HMT)	*qss2*^**-**^	Increased sporulation	Similar to WT with pH control	CRISPR-Cas9	(Feng et al. 2020)
	N1-4(HMT)	*qss3*^**-**^	No sporulation	Similar to WT with pH control	CRISPR-Cas9	(Feng et al. 2020)
	N1-4(HMT)	*qss5*^**-**^	No sporulation	Similar to WT with pH control	CRISPR-Cas9	(Feng et al. 2020)

**Fig. 2:**
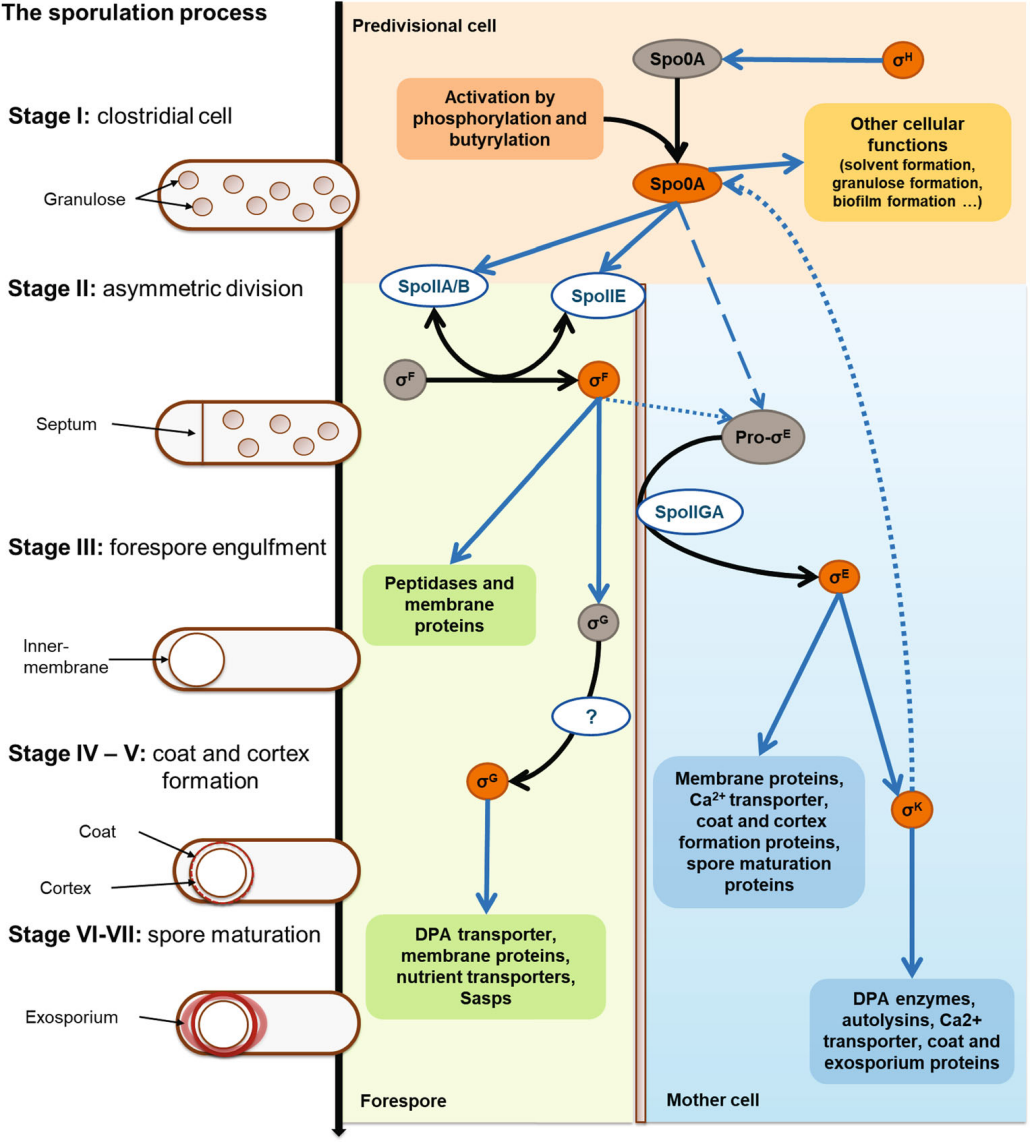
Morphological changes and molecular regulation of sporulation in *C. acetobutylicum* modified from (Al-Hinai et al. 2015). The regulation of the sporulation process is mainly realized by the modulation of the transcription in each compartment. Post-translational regulation enables the activation of Spo0A and sporulation-specific transcription factors (σ^F^, σ^G^, σ^E^). The activation mechanism of σ^G^ has not been investigated in *C. acetobutylicum*. Inactive transcriptional regulators are in grey, and active transcriptional regulators are in orange. DPA, dipicolinic acid; Sasps, small acid-soluble proteins. Black arrows indicate post-translational regulations, blue arrows transcriptional regulation. Arrows with short dashes indicate interactions described only in *C. acetobutylicum*, arrows with long dashes observed only in *C. beijerinckii,* and full arrows indicate interactions described in *C. acetobutylicum* and other clostridia

The regulation model of sporulation in *Clostridium* is divided, like in *Bacillus*, into seven stages associated with morphological changes of the cell. In most clostridia, the sporulation process coincides with granulose accumulation and ends with the lysis of the mother cell and the release of the spores in the environment (Fig. 2). Sporulation is initiated at the end of vegetative growth and is reflected at the transcriptomic level by an increase in *spo0A* expression. Spo0A, the general regulator of the transition from vegetative to stationary growth, is conserved in all *Firmicutes* and has a central role in sporulation, toxin, and solvent production (Al-Hinai et al. 2015; Dürre 2014; Jones et al. 2008; Paredes et al. 2005; Ravagnani et al. 2000; Sauer et al. 1995).

In *Bacillus*, at the end of vegetative growth, AbrB lifts its repression on σ^H^; σ^H^ then promotes the transcription of *spo0A* (Piggot and Hilbert 2004)*.* In solventogenic clostridia, the role of σ^H^ in *spo0A* regulation is unknown. The gene encoding σ^H^ is constitutively expressed in clostridia, which implies that the mechanism occurring in *Clostridium* differs from the one described in *Bacillus*. Several AbrB homologs were identified in solventogenic clostridia, in *C. acetobutylicum* ATCC 824, three homologs (*cac0310*, *cac1941*, and *cac3647*) were disrupted to study their role in the regulation of cellular events. The disruption of the most expressed AbrB homolog, *cac0310*, delayed sporulation and impaired solvent production (Scotcher et al. 2005). The disruption of *cac3647* increased solvent production, while the solvent production of Δ*cac1941* cultures decreased by 6% compared to the wild type (Xue et al. 2016). No change in the sporulation of the Δ*cac1941* or Δ*cac3647* mutant was reported. According to these results, AbrB homologs belong to the sporulation and solvent regulation network in solventogenic clostridia. Unlike in *Bacillus*, these results indicate that AbrB (Cac0310) may promote sporulation in *C. acetobutylicum*. The Spo0A DNA binding motif, called 0A box, was found upstream of *cac0310* and its homolog in *C. beijerinckii* NCIMB 8052, *cbei4885*, indicating that these *abrB* homologs belong to the Spo0A regulon. In fact, the disruption of this 0A box might impair solvent production and sporulation; one of the SNPs detected in asporogenous offspring of *C. beijerinckii* SA-1 (Table 2) was an SNP in the 0A box of *cbei4885* (Seo et al. 2017a).

**Fig. 3:**
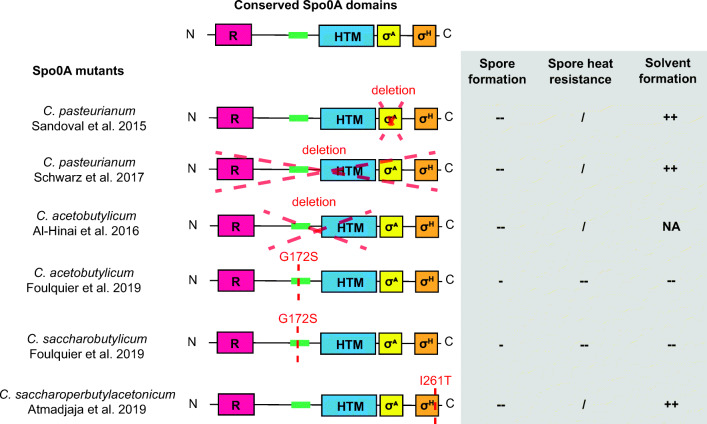
Mutations and associated phenotypes of markerless *spo0A*- strains. Five conserved regions are indicated by colored boxes; R: signal receiver domain, Green box: conserved region with no known function, HTM : helix turn motif, σ^A^: putative σ^A^ activator region, σ^H^: putative σ^H^ activator region. The ability to sporulate, to form heat resistant spores, and to produce solvent is indicated next to the scheme of the *spo0A* mutation present in each mutant strain; - : indicate a decrease compared to the wild type phenotype; -- : indicate the abolition of the feature in the mutant compared to the wild type phenotype; / : indicate that this characteristic could not be evaluated ; ++ indicate an increase compared to the wild type phenotype and NA stands for no data available

Mutations in the *spo0A* coding sequence of solventogenic clostridia have been shown to affect cell physiology considerably. Changes in growth, colony morphology, sporulation, and solvent productivity have been reported. Numerous *spo0A* mutants have been characterized (Table 2), and all have impaired sporulation with sometimes a change in solvent productivity (Atmadjaja et al. 2019; Harris et al. 2002; Seo et al. 2017b). The consequences for the phenotype seem to depend on the mutation location and the studied species. The Spo0A sequence harbors various domains that are conserved among *Firmicutes* and which are putatively involved in sporulation regulation (Fig. 3). Insertional mutations were shown to impair the protein function, blocking the central role of Spo0A in solventogenesis and sporulation regulation (Harris et al. 2002; Wilkinson and Young 1994 ). However, due to the gene engineering techniques used, the impacts of polar effects on the phenotype cannot be excluded (Bayat et al. 2018), but with the development of markerless gene engineering methods, precise mutations of *spo0A* were generated.

Mutations (deletions and single nucleotide modifications) in the putative σ factor activator domains did not cause a decrease in solvent production, as shown in Fig. 3. Like in *Bacillus subtilis* (Schmeisser et al. 2000)*,* a mutation in the putative σ^H^ activator region disrupted the sporulation of *C. saccharoperbutylacetonicum* (I261T). In *Bacillus*, sporulation impairment was explained by a loss in the binding affinity of Spo0A for *spoIIE* and *spoIIA* promoters*.* Mutations in the region upstream from the DNA binding domain impaired solvent production and decreased sporulation efficiency in *C. acetobutylicum* and *C. saccharobutylicum* (Foulquier et al. 2019). In *C. pasteurianum*, a deletion in the σ^A^ activator region disrupted sporulation and increased solvent production (Sandoval et al. 2015). Surprisingly, in *C. pasteurianum*, the deletion of most of the gene (816 bp out of 822) led to the same phenotype (Schwarz et al. 2017). Spo0A seems to play a different role in sporulation regulation in *C. pasteurianum*.

Next to the integrity of the *spo0A* gene, intracellular Spo0A levels appear to also play a role in sporulation regulation. In the non-sporulating strain, *Clostridium* sp. MF28, relatively low *spo0A* expression levels were detected, compared to other solventogenic clostridia, even though high solvent titers could be reached (Li and He 2016). In *C. acetobutylicum*, *spo0A* overexpression increased sporulation independently from the promoter region used (Ehsaan et al. 2016; Harris et al. 2000; Tracy et al. 2008). In contrast, in *C. beijerinckii*, *spo0A* overexpression led to a decrease in both sporulation and solvent production (Kolek et al. 2017). A slight change in Spo0A homeostasis might lead to a different regulation of sporulation and solventogenesis. No detailed study on the variation of active Spo0A and its impact on sporulation has been done in solventogenic clostridia to confirm this hypothesis. In *Bacillus,* depending on the intracellular concentration of phosphorylated Spo0A, differences in the expression of the Spo0A regulon were described (Fujita et al. 2005; Narula et al. 2012).

**Fig. 4:**
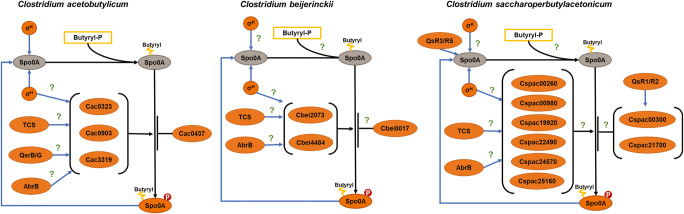
Transcriptional and post-translational regulation of Spo0A in *C. acetobutylicum*, *C. beijerinckii*, *C. saccharoperbutylacetonicum*. Figure adapted from (Al-Hinai et al. 2015) with results from (Feng et al. 2020; Kotte et al. 2020; X. Xin et al. 2020; J.-Y. Xu et al. 2018; Y. Yang et al. 2020). Blue arrows transcriptional regulation and black arrows post-translational regulation. The interrogation marks indicate interactions remain to be proven experimentally

Once *spo0A* is transcribed and translated, Spo0A is activated by at least two post-translational modifications: acylation and phosphorylation, as illustrated in Fig. 4. Protein acylation is a post-translational modification that consists in adding an acyl group to a lysine residue. This reaction is reversible and does not need the intervention of an enzyme. Acylation neutralizes the negative charge of the lysine residue, altering the protein structure and its interaction with other proteins, cofactors, or substrates (Macek et al. 2019). Interestingly, Spo0A acylation was only reported in *C. acetobutylicum* (Xu et al. 2018). In *Bacillus*, acylation was also linked with sporulation (Kosono et al. 2015), but it was observed only for late-sporulation stage proteins (CotE, CotO, Cse15, SpoIVD, and SpoVR). Xu et al. showed in their study in *C. acetobutylicum* that several key proteins involved in metabolism and life cycle, such as Buk and Spo0A, were butyrylated during cultivation (Xu et al. 2018). Two butyrylation sites were detected in the Spo0A sequence, one close to the phosphorylated domain and another on the DNA binding domain, and replacing the lysine residue (K217) located in the DNA binding domain with glutamine decreased the DNA binding affinity of Spo0A. While the wild-type Spo0A could bind to its binding motif in the promoter region of Spo0A, the mutated Spo0A could not. This result suggests that Spo0A butyrylation is necessary for Spo0A activity and its autoregulation.

In *Clostridium*, the phosphorylation mechanism activating Spo0A also contrasts with the phosphorelay system described in *B. subtilis* (Paredes et al. 2005), in that it relies on orphan histidine kinases, as illustrated in Fig. 4. Orphan histidine kinases are part of two-component quorum sensing systems (TCS). TCS usually consists of a sensor histidine kinase and a response regulator located in the same operon. However, isolated genes encoding sensor histidine kinases or response regulators were identified in the genome of numerous bacteria and especially in *Firmicutes*. Since these genes are not co-located with a gene encoding a specific sensor protein or response regulator, they are labeled orphans (Davidson et al. 2018; Williams and Whitworth 2010). Unlike regular TCS kinases, orphan histidine kinases can modulate several response regulators. In clostridia, orphan histidine kinases are integrated for signal transduction to the sporulation regulation network (Freedman et al. 2019; Paredes-Sabja et al. 2014; Steiner et al. 2011; X. Xin et al. 2020). In *C. acetobutylicum*, the impact of four histidine kinases (Cac0323, Cac0437, Cac0903 and Cac3319) on sporulation has been studied (Steiner et al. 2011). In vitro studies proved that two of these kinases, Cac0903 and Cac3319, are able to activate Spo0A. The disruption of *cac0323*, *cac0903*, and *cac3319* resulted in a decreased sporulation, in contrast to *cac0437* deficient strains hyper-sporulated (earlier sporulation and a fifteen-fold increase in heat resistant spores). In *C. beijerinckii* NCIM 8052, six homologs of these kinases were disrupted in a recent study (Xin et al. 2020). A significant decrease in sporulation efficiency (between 70 and 90%) coupled with an increase in solvent production of 38% and 14% were reported only for the *∆cbei2073* and *∆cbei4484* mutants, respectively*.* Interactions between these histidine kinases and Spo0A remain to be studied. Recently Seo et al. analyzed the genome of the hyper-butanol-producing mutant BA101 strain and identified mutations in the genome that could explain its reduced sporulation (Seo et al. 2021). Two mutations could explain their asporogenous phenotype; one in a gene encoding a histidine kinase (*cbei_3078*) and a second mutation in a gene coding for a serine/threonine protein phosphatase-like protein (*cbei_4400*). Similarly, the mutation in *cbei_4400* was also reported in SA-1, another hyper-producing *C. beijerinckii* strain with delayed sporulation (Sandoval-Espinola et al. 2013).

Once phosphorylated, Spo0A binds to the 0A box to regulate the expression of sporulation- and solvent genes (Ravagnani et al. 2000; Zhao et al. 2002). Differences in the motif sequence (Ravagnani et al. 2000), the number (Sullivan and Bennett 2006), and the location of these 0A boxes (Patakova et al. 2013) between solventogenic clostridia were detected. These discrepancies further illustrate the variations in the role of Spo0A in solventogenic clostridia.

#### Stages of sporulation

In Fig. 2, a scheme of the stages of sporulation and phenotypic changes during sporulation is shown. Stage I of the sporulation process starts with the DNA replication and the positioning of Z-rings, close to the poles, to prepare for asymmetric division (Barák et al. 2019). Solvent production is initiated, and in most clostridia, Stage I coincides with a morphological change. The cell swells due to the accumulation of a starch-like polymer called granulose. In *C. acetobutylicum*, granulose and sporulation are regulated by an Agr quorum system (Steiner et al. 2012). In other solventogenic clostridia, only one study links granulose production and sporulation (Ravagnani et al. 2000). While investigating the role of Spo0A in *C. beijerinckii*, Ravagnani et al. discovered that Spo0A was essential for the accumulation of granulose. Still, granulose accumulation was not described in all solventogenic clostridia; *C. tetanomorphum*, for example, does not produce any granulose during the sporulation process (Patakova et al. 2014).

Once activated, Spo0A promotes the expression of *spoIIE*, triggering the entrance in stage II of the sporulation process. During Stage II, a septum forms on one pole of the cell dividing it into two compartments, the forespore and the mother cell. In *Bacillus*, this asymmetric division is orchestrated by SpoIIE and the cell division proteins (involved in binary fission) (Barák et al. 2019). SpoIIE simultaneously activates σ^F^, the first sporulation-specific sigma factor, which is kept inactive by SpoIIAB. SpoIIE phosphorylates SpoIIAA, which binds the anti-sigma factor SpoIIAB which then releases σ^F^ in the forespore. In solventogenic clostridia, the role of SpoIIE was studied in *C. acetobutylicum* and *C. beijerinckii* through the generation of SpoIIE deficient mutants (Bi et al. 2011; Diallo et al. 2020b; Scotcher and Bennett 2005). Both mutants could no longer sporulate, but phenotypical differences between *C. beijerinckii ΔspoIIE* and *C. acetobutylicum ΔspoIIE* were noted. In *C. acetobutylicum*, *spoIIE* disruption prevented the formation of an asymmetric septum, while in *C. beijerinckii,* misplaced septa were observed. The morphology of the *C. beijerinckii* mutant corresponded to the *spoIIE* mutants described for *Bacillus*. This discordance in the mutants’ morphology indicates differences in the asymmetric septation mechanism of solventogenic clostridia. Another difference was observed; no critical change in the expression of *sigF* and *sigE* was detected in *C. beijerinckii ΔspoIIE,* contrasting with *C. acetobutylicum ΔspoIIE,* where *sigF* and *sigE* were downregulated.

The completion of asymmetric division marks the entrance in Stage III, during which the septum is prolonged and surrounds the whole forespore. This phenomenon, called engulfment, yields an isolated compartment surrounded by two membranes within the mother cell. Engulfment is coordinated by proteins belonging to σ^F^ (in the forespore) and σ^E^ regulons (in the mother cell). The roles of σ^F^ and σ^E^ were studied only in *C. acetobutylicum* ATCC 824, where *sigF* and *sigE* mutants were generated. Once again, these mutants’ cell morphology did not correspond to the morphology of *sigF* and *sigE* mutants generated in *Bacillus* or other clostridia (Al-Hinai et al. 2015). In both *C. acetobutylicum* mutants, the sporulation process was interrupted before the formation of the asymmetric septum, as observed for the *spoIIE* mutant. This suggests an earlier function of σ^F^ and σ^E^ in sporulation regulation (Jones et al. 2011; Tracy et al. 2011). The disruption of either *sigF* or *sigE* affected solventogenesis. Indeed, when mid to late exponential cells were inoculated for fermentation, solvent production decreased significantly.

After engulfment, the coat and the spore cortex are formed during Stage IV and V. Dipicolinic acid (DPA) is produced in the mother cell through the conversion of aspartate, and then transported into the forespore, in exchange for water, to bind Ca^2+^ in the forespore (Piggot and Hilbert 2004). Ca^2+^-DPA attaches to the forespore’s DNA to protect it against heat damages (Jamroskovic et al. 2016; Paidhungat et al. 2000; Paredes-Sabja et al. 2008). In the meantime, coat proteins assemble around the mother-cell-derived membrane of the forespore (Shen et al. 2019). All these events are coordinated by proteins regulated by σ^G^ in the prespore and σ^K^ in the mother cell. In contrast to pathogenic clostridia, these late stages of sporulation are barely studied in solventogenic clostridia. Nonetheless, σ^K^ and σ^G^ deficient mutants in *C. acetobutylicum* confirmed their crucial role in sporulation (Al-Hinai et al. 2014; Tracy et al. 2011). Disruption of *sigG* interrupted sporulation after engulfment as described in *Bacillus* and did not affect solventogenesis. The disruption of *sigK*, though, did not yield the same phenotype as the *Bacillus* mutant. In *C. acetobutylicum,* σ^K^ regulates sporulation initiation and sporulation maturation, as described in other clostridia (Al-Hinai et al. 2015). No Spo0A proteins were detected in *sigK* mutants of *C. acetobutylicum*, and the introduction of an extrachromosomal copy of *spo0A* under control of the *ptb* promoter led to the formation of heat-sensitive spores.

During Stages VI and VII, the spore matures as the size of both cortex and coat increases. Sporulation finishes with the lysis of the mother cell and the release of the spore in the environment. A study on an autolysin deficient mutant, *C. acetobutylicum lyc::int(72)*, generated in *C. acetobutylicum* ATCC 824*,* showed that autolysins are needed to complete sporulation (Liu et al. 2015). The number of viable spores produced by *C. acetobutylicum lyc::int(72)* decreased by 30% compared to the wild-type strain. According to the authors, cell lysis might provide additional nutrients to sporulating cells and thus be required for successful sporulation.

Only a few studies on the molecular regulation of sporulation in solventogenic clostridia have been published (Al-Hinai et al. 2014; Bi et al. 2011; Diallo et al. 2020b; Jones et al. 2008, 2011; Ravagnani et al. 2000; Scotcher et al. 2005; Scotcher and Bennett 2005; Steiner et al. 2011, 2012; Tracy et al. 2011). Studies were mainly realized in *C. acetobutylicum* ATCC 824, and studies with other solventogenic strains show variations in the role of Spo0A and SpoIIE in the regulation of sporulation. Moreover, the post-translational regulation for sigma factor activation, observed in *Bacillus* and *Clostridioides* (former *Clostridium*) *difficile*, still needs to be investigated in solventogenic clostridia. Thus, several grey areas remain concerning the regulatory mechanisms controlling the sporulation cascade in solventogenic clostridia.

#### The clostridial endospore

**Fig. 5:**
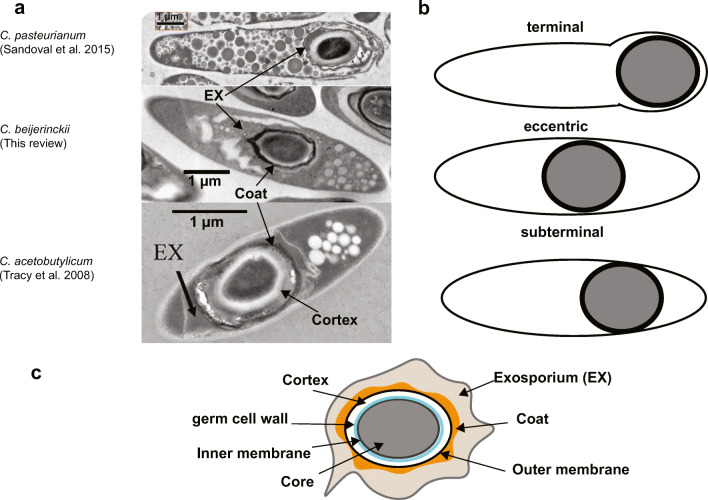
Morphology and composition of the clostridial endospore; **a** Transmission electron micrographs of mature endospores from three solventogenic clostridia. Depending on the species, the size of the cortex, as well as the location of the endospore in the mother cell, changes; **b** Possible location of the endospore in solventogenic clostridia; **c** Composition of the endospore. EX stands for exosporium

At the end of sporulation, an endospore is released into the environment. The endospore is highly dehydrated and organized in proteinous layers protecting the core, which hosts the DNA. Five layers surround the core: the inner membrane, the germ cell wall, the cortex, the outer membrane, and the coat (Fig. 5b). These layers ensure a robust protection of the core against chemicals, oxygen, enzymes, and heat. In the core, the DNA is bound to small acid-soluble proteins (Sasps), ribosomes, enzymes, and DPA. The DPA content can reach up to 25% of the spore’s dry weight (Paredes-Sabja et al. 2014).

Spores are characterized by their size, shape, and location in cells (Dürre 2005, 2014). There is not a typical morphology for all clostridial spores, and few studies have been done to compare the spore morphology of clostridial strains. One study (Berezina et al. 2012) compared the spore morphology of the four main solventogenic species (*C. acetobutylicum*, *C. beijerinckii*, *C. saccharobutylicum,* and *C. saccharoperbutylacetonicum*) and showed that they all possess an oval shape. This characteristic cannot be generalized to all solventogenic clostridia since *C. tetanomorphum* yields round-shape spores (Patakova et al. 2014). The spore’s location may vary within the mother cell (Fig. 5a, b) from eccentric to terminal (Dürre 2005).

The spore coat (Long et al. 1983), the cortex, and even the core composition are species- and even sometimes strain-specific. For instance, differences in the spore’s DPA and Ca^2+^ content were detected, resulting in a difference in heat resistance and that the Ca^2+^/DPA ratio was not species but strain-specific (Jamroskovic et al. 2016) showed that a high Ca^2+^/DPA ratio correlates with more resistant spores. *C. acetobutylicum* ATCC 824 spores can sustain a long heat treatment (> 5 min) at 70 ^o^C, while *C. beijerinckii* NCIMB 8052 spores germinate better with a short heat treatment (1 min) at higher temperatures (around 90 ^o^C). The heat-shock treatment has to be adapted to the species to give the highest germination efficiency. Knowledge of these differences is crucial as it affects germination efficiency after a heat-shock treatment before fermentation (Jabbari et al. 2013; Li et al. 2011; Steiner et al. 2012).

### Sporulation triggers

The sporulation is known to be a response to stressful conditions. The primary triggers of sporulation in *Bacillus* species have been shown to be nutrient starvation and high cell density (Awang et al. 1992; Basu et al. 2017). In contrast to bacilli, where the cessation of growth occurs due to a lack of nutrients, in *Clostridium*, an excess of a carbon or nitrogen source led to growth cessation (Dürre 2005; U. Sauer et al. 1995; Woods and Jones 1987). Furthermore, unlike bacilli, clostridia are anaerobic bacteria; thus, oxygen is a significant stress factor for the cells and triggers sporulation.

Several studies (Awang et al. 1992; Kolek et al. 2017; Long et al. 1983; Sedlar et al. 2021; Woods and Jones 1987) have reported the substantial impact of medium composition on sporulation initiation and sporulation efficiency (number of spore generated and the spores that can germinate) and suggested a link between carbon source, mineral content, external pH, and the number of spores produced.

#### The carbon source

Solventogenic clostridia can ferment several carbohydrates (including C6 and C5 sugars and sugar polymers such as starch or xylan), yet, the solvent yield varies depending on the carbon source (Awang et al. 1992; Shaheen et al. 2000). Similarly, sporulation efficiency depends on the carbon source, as described for *C. saccharobutylicum* NCP 262 (Long et al. 1983). A complementary study with the same strain assessed the effect of 13 different carbon sources (carboxymethylcellulose, xylan, inulin, a starch/glucose mix, lactose, cellobiose, sucrose, maltose, glucose, mannose, fructose, galactose, and xylose) on both sporulation and solvent formation (Awang et al. 1992). Depending on the carbohydrate, sporulation frequency varied up to 44%, with glucose utilization leading to the most spores. Likewise, when grown on rhamnose, a decrease in spore formation was observed in *C. beijerinckii* cultures (Diallo et al. 2018). Recently Sedlar et al. observed that *C. beijerinckii* NCIMB 8052 and *C. beijerinckii* NRRLB 598 sporulated when glycerol was added to the medium instead of glucose (Sedlar et al. 2021). Surprisingly the opposite was observed in *C. beijerinckii* (formerly *C. diolis*) DSM 15410 cultures. The substrate may also accelerate sporulation initiation. For instance, xylose-fed cultures of *C. acetobutylicum* BOH3 sporulated earlier than glucose-fed cultures (Basu et al. 2017). In addition to the nature of the carbohydrate, its concentration also affects sporulation. High glucose concentrations doubled the number of endospores generated by *C. saccharobutylicum* NCP 262 (Long et al. 1984a).

These observations have been confirmed by studies on carbon metabolite repression in clostridia. The carbon catabolite repression protein A (CcpA) regulates the carbon catabolite repression in *Firmicutes* and is involved in triggering sporulation in pathogenic clostridia. CcpA activates (Varga et al. 2004) or represses sporulation (Antunes et al. 2011) depending on the species. Still, in *C. acetobutylicum,* CcpA positively regulates sporulation (Ren et al. 2012). In a CcpA deficient strain, sporulation was delayed, and the sporulation efficiency decreased (Table 2). Besides, transcriptome analysis of the mutant strain performed by Ren et al. suggested that CcpA represses *abrB* homologs and promotes the expression of the sporulation-specific sigma factors (*sigE*, *sigG*, *sigK*). This modification of the expression profile of those sporulation-related genes might have altered sporulation in the CcpA deficient *C. acetobutylicum* strain.

#### Other media components

Solventogenic clostridia are currently investigated for their potential to produce solvents from complex feedstock such a lignocellulosic and algal feedstocks. Pretreatment of these feedstocks is necessary to the utilization by the bacteria of the carbohydrates present. During the pretreatment, di- and monosaccharides, as well as inhibitory chemicals (salts, furfurans and phenolic compounds), are formed. Studies have been conducted to evaluate the impact of these toxic compounds on cell growth and solvent formation. Hence, few reports on their effect on sporulation can be found; still, three transcriptomic studies of *C. beijerinckii* and *C. acetobutylicum* cultures exposed to phenolic compounds detected changes in the expression of sporulation genes. Exposure to ferulic acid (Lee et al. 2015) and syringaldehyde (Ezeji et al. 2007) caused an upregulation of the late-stage sporulation genes *C. beijerinckii*. In *C. acetobutylicum*, a recent study showed through a gene coexpression network analysis (Liu et al. 2020) that exposure to vanillin and p-coumaric acid disturbed the transcription of early sporulation genes (*spo0A, spoIIE, spoIIP*) and sporulation specific sigma factors.

Acids, various metals and minerals, vitamins, and amino acids also affect both solvent production and sporulation in clostridia (List et al. 2019; Mukherjee et al. 2019; Nimbalkar et al. 2018, 2019; Reeve and Reid 2016), but few studies mentioned their impact on sporulation. Long et al. (1984a) investigated butyrate and acetate’s effect on sporulation in *C. saccharobutylicum* by adding them to the medium at the start of the fermentation in different concentrations. Even though the addition of acids was not necessary for sporulation, it increased the number of spores present in the culture by 40 to 100% for concentrations between 1 and 4 g L^-1^.

As for the impact of other media components on sporulation, one study reports that the addition of adenine in the media caused a 20-h delay in the onset of sporulation in *C. saccharoperbutylacetonicum* cultures (Kiyoshi et al. 2017). It is worth noting that, depending on the species, a compound can have an opposite impact on sporulation. For example, in *C. perfringens*, iron is necessary for sporulation (Lee et al. 1978), while its addition to the medium impairs sporulation in *C. sporogenes* (Mah et al. 2008).

Solventogenic clostridia harbor sporulation proteins requiring metal-containing cofactors; thus, the media’s metal content is expected to impact sporulation regulation. For instance, homologs of SpoIIE, SpoIIQ and CsfB, an anti-sigma factor of σ^E^ and σ^G^, were identified in *Clostridium*. In *Bacillus*, their activity requires Mn^2+^and Zn^2+^ respectively (Król et al. 2017; Martínez-Lumbreras et al. 2018). In *Bacillus,* Mn^2+^ was proven to be crucial for SpoIIE’s phosphatase activity and the oligomerization of SpoIIE, and thus, asymmetric division (Król et al. 2017). Mn^2+^ has also been reported to be key for the development of heat-resistant spores in *C. botulinum* (Lenz and Vogel 2014). In *C. difficile*, Zn^2+^ is necessary for the formation of the SpoIIQ-SpoIIIAH complex, involved in engulfment and essential for the transit of molecules between mother cell and forespore (Serrano et al. 2016). Zn^2+^ was reported to promote sporulation in *C. botulinum* (Kihm et al. 1988) but to inhibit sporulation of *C. sporogenes* when the concentration in the medium exceeds 3.7 mM. (Lee et al. 2011). Ca^2+^ is also involved in spore formation since it forms together with dipicolinic acid (DPA), and several studies have shown that Ca^2+^is a crucial component for spore heat resistance (Church 1959; Huang et al. 2007; Jamroskovic et al. 2016; Mah et al. 2008; Paredes-Sabja et al. 2008).

Studies of the transcriptome of wild-type and mutant *C. beijerinckii* cultures during fermentation indicated changes in the expression of genes involved in ion- and amino acid transport at sporulation initiation. In *C. beijerinckii* NRRL B598, sporulation initiation was concomitant with an upregulation of the genes encoding a magnesium transporter, and an upregulation of genes encoding potassium, sodium, and iron transporters was detected during stationary phase (Vasylkivska et al. 2019). In cultures of the asporogenous *C. beijerinckii* Δ*spoIIE* strain, the expression of genes encoding iron transporters were downregulated during stationary phase (Diallo et al. 2020b), indicating a potential role of iron in sporulation.

#### Metabolite concentration

**Fig. 6:**
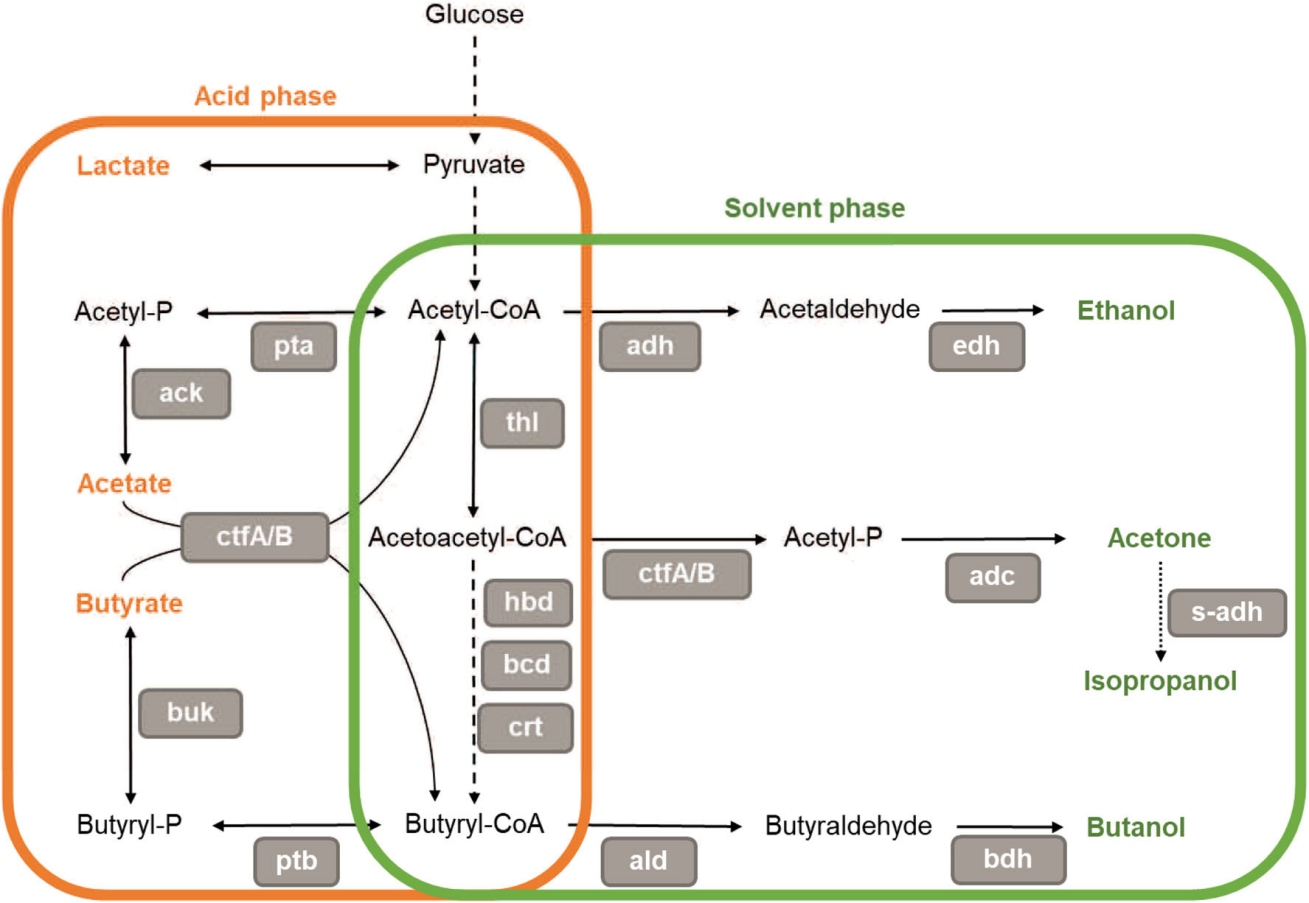
Simplified acetone-butanol-ethanol metabolic pathway in solventogenic clostridia. Some strains harbor a secondary alcohol dehydrogenase (s-adh) that enables the formation of isopropanol. In *C. acetobutylicum*, the acidogenic phase and solventogenic phase succeed each other during the fermentation. During exponential growth, the substrate is metabolized to form lactate, acetate, and butyrate during the acid phase. At stationary phase, the acids are reassimilated, and the culture produces ethanol, acetone (or isopropanol), and butanol. However, in other solventogenic species (*C. beijerinckii* for example), acidogenic and solventogenic phase occur concomitantly and solvent production starts during the exponential phase. The enzymes involved in the metabolic pathway are in grey boxes: pta, phosphotransacetylase; ack, acetate kinase; thl, thiolase; hbd, 3-hydroxybutyryl-CoA-dehydrogenase; crt, crotonase; bcd, butyryl-CoA-dehydrogenase; ctfA/B, CoA-transferase; buk, butyrate kinase; ptb, phosphotransbutyrylase; adh, aldehyde/alcohol dehydrogenase; edh, ethanol dehydrogenase; adc, acetoacetate decarboxylase; s-adh, secondary alcohol dehydrogenase; ald, butyraldehyde dehydrogenase; bdh, butanol dehydrogenase and -P stands for phosphate. As indicated by its title, this figure represents a simplified ABE pathway; indeed, studies have shown a diversity in the structure and number of enzymes involved depending on the solventogenic species

Metabolite stress has been suggested to trigger sporulation in solventogenic clostridia (Heluane et al. 2011; Sauer et al. 1995; Tomas et al. 2004; Zheng et al. 2009). When grown in batch reactors, solventogenic clostridia ferment the available carbohydrates into carboxylic acids, mainly acetate and butyrate, which accumulate in the culture and cause a drop in pH (Fig. 6).

At the onset of solventogenesis and sporulation, acids are reassimilated and converted into solvents, resulting in a rise of pH in the culture. While solvent formation enables short-term relief from the pH stress, sporulation is regarded as a long-term stress response mechanism, protecting the cells from metabolic stress and interrupting sugar degradation (U. Sauer et al. 1995). Acetate and butyrate accumulation during exponential growth is proposed to trigger both solventogenesis and sporulation (Sauer et al. 1995; Thorn et al. 2013). In *C. acetobutylicum*, a peak in the intracellular undissociated acid concentration was observed at the start of the solventogenesis (Terracciano and Kashket 1986; Yang et al. 2013). Characterization of *C. acetobutylicum* recombinants deficient in phosphotransbutyrylase (Ptb), butyrate kinase (Buk) and acetate kinase (Ack) (Desai and Papoutsakis 1999; Green et al. 1996; Harris et al. 2000; Kuit et al. 2012) suggested that instead of the concentration of undissociated acids, the intracellular concentration of butyryl phosphate (BuP) might trigger both sporulation and solventogenesis. BuP is an intermediary metabolite in the ABE metabolic pathway (Fig. 6), formed during acidogenesis during butyrate formation. Studies evaluating the intracellular concentration of BuP during batch cultivation showed that BuP was indeed accumulated in the cell (Xu et al. 2018; Zhao et al. 2005). Two peaks in the cytoplasmic BuP concentration were detected inside the cells, one at the beginning of the cultivation and a second one coinciding with solventogenesis and sporulation initiation (Y. Zhao et al. 2005). It was suggested that BuP acted as a phosphate donor enabling the activation of Spo0A, the master regulator of sporulation and solventogenesis (Kuit 2013; Zhao et al. 2005), but recent data (Xu et al. 2018) present another post-translational regulation mechanism: protein butyrylation (see section on the initiation of sporulation).

Butanol has also been suspected of triggering sporulation (Zheng et al. 2009). Even though solventogenic clostridia naturally produce butanol, it affects cell growth when its concentration exceeds 0.5% v/v in the culture (Sedlar et al. 2018) and becomes lethal, around 1.5% v/v (Sedlar et al. 2019). Butanol concentration being a stress factor for the cells, researchers supposed that a rise in butanol concentration would initiate sporulation before it reaches toxic concentration. However, a decrease in granulose and spore number was observed in butanol stressed *C. beijerinckii* cultures (Sedlar et al. 2019). Transcriptional studies on butanol stressed cultures of *C. acetobutylicum* and *C. beijerinckii* showed no notable changes in the expression of the genes encoding the sporulation-specific sigma factors (*sigF*, *sigE*, *sigG*, *sigK*) (Patakova et al. 2019; Tomas et al. 2004). In *C. acetobutylicum* ATCC 824*,* no butanol-dependent impact on sporulation efficiency was described. Instead, a decrease in the expression of genes encoding small acid-soluble proteins was observed (Schwarz et al. 2012; Tomas et al. 2004). These proteins protect the DNA present in the spores and are crucial for their heat resistance (Leggett et al. 2012).

Secondary metabolites have also been reported to promote sporulation in solventogenic clostridia. Two categories of secondary metabolite biosynthesis gene clusters were identified in solventogenic clostridia (Letzel et al. 2013), polyketide- and ranthipeptide biosynthesis clusters. Polyketides have been studied in *C. acetobutylicum and C. saccharoperbutylacetonicum*. In *C. saccharoperbutylacetonicum,* polyketides involved in sporulation initiation, solvent formation, and tolerance were detected (Kosaka et al. 2007; Li et al. 2020a). In *C. acetobutylicum*, three polyketides were detected, and the structures of two of them, clostrienose and clostrienoic acid, were solved (Herman et al. 2017). In both species, the disruption of polyketide clusters decreased sporulation (Table 2). In *C. beijerinckii*, polyketides might also intervene in the regulation of sporulation; the interruption of sporulation in *C. beijerinckii* Δ*spoIIE* affected the expression of the polyketide gene cluster (Diallo et al. 2020b). Recently, the role of ranthipeptides, secondary metabolites belonging to the ribosomally synthesized and post-translationally modified peptide (RiPP) superfamily, was studied in *C. beijerinckii* and *C. ljungdahlii* (Chen et al. 2020). In *C. beijerinckii*, the genes encoding the precursor peptide and the radical SAM protein were disrupted, and the impact on the transcriptome was evaluated by RNA sequencing. In the mutant strain, sporulation genes were strongly downregulated and the *agr* locus encoding the Agr quorum sensing mechanism was upregulated. Secondary metabolites seem to play an important role in the initiation of sporulation, even so the interactions between the polyketides and ranthipeptides with sporulation regulators remain to be investigated.

#### Quorum sensing and cell density

**Fig. 7:**
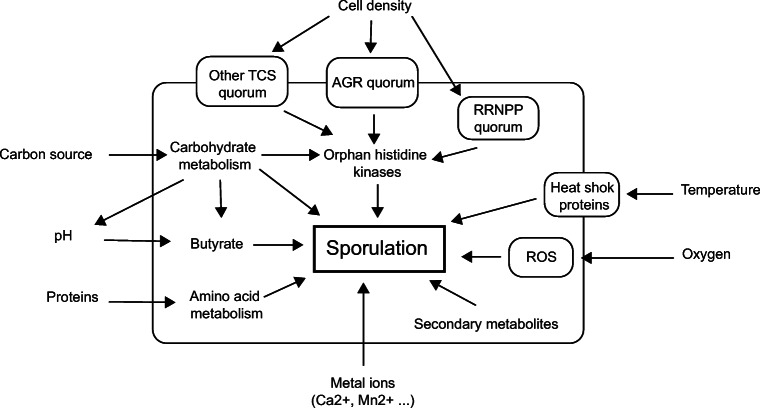
Triggers of sporulation in solventogenic clostridia and the signal transduction systems involved in sporulation regulation; TCS, two-component system family; RRNPP, Rgg/Rap/ NprR/PlcR/PrgX quorum system family; ROS, reactive oxygen species-dependent mechanisms

As described for *B. subtilis* (Bischofs et al. 2009; Grossman and Losick 1988), cell density might regulate sporulation in solventogenic clostridia. In *Bacillus*, a minimum cell density was required for efficient sporulation (Grossman and Losick 1988; Hecker and Völker 2001). Similarly, in continuous cultures of *Clostridium*, where the specific dilution rate and cell morphology can be monitored, a decrease in the number of sporulating cells was observed when the dilution rate was raised (Heluane et al. 2011). Thanks to quorum sensing mechanisms, cells can monitor environmental changes such as cell density and launch their adaptation response when required. Two quorum sensing mechanism superfamilies were described in Gram-positive bacteria (Aframian and Eldar 2020): the membrane receptor family (TCS) and the cytoplasmic receptor family (RRNPP). Systems belonging to both families were found in solventogenic clostridia (Fig. 7). Two TCS, an Agr system and the BtrK/BtrR system, were described in *C. acetobutylicum* ATCC 824 (Steiner et al. 2012; Yang et al. 2020)*.* The Agr system regulates both granulose formation and sporulation. In contrast, the BrtK/BtrR system seems to detect other environmental changes and regulates the growth rate, the start of solventogenesis and butanol tolerance. No role in sporulation regulation was described; still, the overexpression of the *BtrK/BtrR* operon changed the expression of genes involved in sporulation initiation (*spo0J*, *spoIIE*, *spoIIR*) and spore maturation (*sigK*, *spoIVA*).

RRNPP quorum-sensing systems positively regulating sporulation were recently identified in *C. acetobutylicum* (Kotte et al. 2020) and *C. saccharoperbutylacetonicum* (Feng et al. 2020). These systems named Qss are composed of two proteins: the receptor Qsr, which harbors a helix-turn-helix region (HTH) common to DNA binding domains, and the signal peptide precursor Qsp. In the genome of *C. acetobutylicum,* eight putative RRNPP systems were detected, but only two seem to intervene in sporulation regulation (Table 2). In *C. saccharoperbutylacetonicum*, four out of the five identified systems affected sporulation. The deletion of *qssR1* and *qssR2* increased sporulation efficiency, while sporulation frequency decreased in Δ*qssR3* and Δ*qssR5* mutants. Moreover, *spo0A* expression decreased in Δ*qssR3* and Δ*qssR5* mutants and *qssR1/2* affected the expression of *spo0E-like* genes*,* putatively involved in the activation of Spo0A*.* However, no regulation mechanism was described in any of these species. In *C. difficile,* a similar RRNPP system (RstA) was described. RstA was reported to modulate the expression of sporulation genes through its DNA binding domain (Edwards et al. 2020). A similar regulation mechanism might take place in solventogenic clostridia. Changes in process parameters such as temperature or dilution rate also appear to trigger sporulation (Heluane et al. 2011; Kiyoshi et al. 2017). Sporulation is initiated by a considerable number of triggers (Fig. 7), and several quorum-sensing mechanisms and messengers interact with transcriptional regulators to integrate all the environmental cues and induce a cellular response.

## Sporulation in another industrially relevant group: the acetogens

Acetogenic bacteria are C-1 compounds utilizing microorganisms. They can fix CO_2,_ CO, or formate to produce acetate through the Wood-Ljungdahl pathway. Because of their unique metabolism and the development of biobased processes for chemical production, acetogens caught the interest of the biotechnological industry. Anaerobic acetogens are particularly interesting for industrial use since anaerobic conditions reduce flammability concerns linked to the use of CO and H_2_ and contamination risks (Liew et al. 2016). Fourteen acetogenic species have been identified within the *Clostridium* genus, and among which six (*C. ljungdahlii, C. coskatii, C. drakei, C. carboxidivorans, C. ragsdalei, C. autoethanogenum*) are also natural solvent producers. Numerous studies on these clostridial acetogens have been done in the last ten years and one company, Lanzatech, managed to scale up its gas fermenting process based on CO conversion to ethanol and operates a commercial plant (LanzaTech 2019).

Few data are available on the sporulation mechanism in acetogens. In fact, sporulation was rarely observed in the cultures of acetogenic clostridia. Still, some brief descriptions of the morphology of sporulating cells can be found for *C. coskatii, C. drakei*, *C. carboxidivorans, C. ragsdalei*, and *C. autoethanogenum* (Abrini et al. 1994; Huhnke et al. 2008; Liou et al. 2005; Zahn and Saxena 2011). As described for solventogenic clostridia, these species form sub-terminal and terminal spores. During sporulation, swelling of the cells was reported in *C. coskatii* (Zahn and Saxena 2011) and *C. carboxidivorans* (Liou et al. 2005)*,* as described for other solventogenic clostridia. In contrast, no swelling of *C. autoethanogenum* (Abrini et al. 1994) and *C. ragsdalei* (Huhnke et al. 2008) cells was observed. A significant difference to most solventogenic clostridia is the absence of granulose in sporulating cells of acetogens.

To verify whether the reduced sporulation was due to a difference in the set of sporulation genes, we performed a blastp analysis (http://blast.ncbi.nlm.nih.gov/Blast.cgi) based on the minimal set of sporulation genes defined by Galperin et al. (Galperin et al. 2012). Homologs to the 52 core sporulation genes were found in the above-mentioned solvent producing acetogens. Further, we searched for homologs of genes known to be involved in the initiation of sporulation in solventogenic clostridia. In all the genomes analyzed, homologs of the Agr and RRNPP quorum-sensing systems, as well as homologs of orphan kinases, were identified. As for secondary metabolites, no polyketide encoding gene cluster (Letzel et al. 2013) was found; still, all strains harbor ranthipeptide producing enzymes. A recent study on the ranthipeptides produced by *C. beijerinckii* and *C. ljungdahlii* highlighted their importance in regulating cellular events (Chen et al. 2020); still, no link with sporulation in *C. ljungdahlii* could be made. Interestingly, homologs to the enzymes involved in granulose formation were found in *C. drakei* and *C. carboxidivorans*, but not in *C. ljungdahlii*, *C. autoethanogenum* or *C. coskatii*.

Therefore, it seems that the low sporulation frequency observed in acetogens is not due to the absence of sporulation genes in the genome. Differences in triggers, transcriptional or post-transcriptional regulations of sporulation proteins during fermentation could explain this low frequency. Using transcriptome analysis of *C. ljungdahlii* and *C. autoethanogenum* generated by Philips et al. 2017, Aklujkar et al. 2017, Whitham et al. 2015 and Diender et al. 2019, we looked at the expression profile of sporulation gene homologs under various conditions. When grown under salt stress conditions, *C. ljungdahlii* did not form spores despite the upregulation of *spo0A* (Philips et al. 2017). An interesting study on the variation of the transcriptome of *C. ljungdahlii* under lithotrophic and organotrophic growth conditions (CO_2_/H_2_ vs. fructose) reported the upregulation of an *agr* homolog and nine σ^E^ regulated sporulation genes during lithotrophic growth (Aklujkar et al. 2017). Surprisingly, no change in *spo0A* expression was observed. A similar change was observed when *C. ljungdahlii* was exposed to oxygen (Whitham et al. 2015). The homologs of *abrB*, *sigE,* and three late-stage sporulation genes were upregulated in the O_2_ challenged cultures. These results show that sporulation genes are expressed during the fermentation in *C. ljungdahlii* and that their expression varied in response to the environmental changes (changes in carbon source and presence of oxygen) as observed in other clostridia. A similar observation was made when studying the transcriptome analysis done on *C. autoethanogenum* (Diender et al. 2019). Diender et al. studied variations of the transcriptome of *C. autoethanogenum* in CO/H_2_ compared to CO grown-cultures and in mono-culture compared to co-culture with *C. kluyveri*. In CO/H_2_ grown-cultures, several stage II and III sporulation genes were upregulated, and a slight increase in the expression of the sporulation-specific sigma factors was detected. In the synthetic co-cultures, an up-regulation of *spo0A* and *spoIIE* homologs was observed, while *sigF* and some sporulation genes involved in cortex formation were down-regulated. According to these results, the regulation of the sporulation in *C. ljungdahlii* and *C. autoethanogenum* seems to have common characteristics with the systems described in other solventogenic clostridia. Like in solventogenic clostridia, carbon sources impact sporulation regulation in *C. ljungdahlii* and *C. autoethanogenum.* Indeed, their growth in lithotrophic conditions led to an upregulation of sporulation genes compared to organotrophic conditions. Moreover, the *sigE* and *sigF* regulons that have been described in *C. acetobutylicum and C. beijerinckii* seem to be conserved in *C. ljungdahlii* and *C. autoethanogenum*. Even so, these results are still not sufficient to claim that the regulatory network governing sporulation is identical in both acetogens and solventogenic clostridia.

## Spore formation in fermentation: enemy or ally?

### Connections between sporulation and solventogenesis

In solventogenic clostridia, sporulation and solvent production are both stress responses to unfavorable environmental conditions. Solventogenesis is thought to be triggered before sporulation (Patakova et al. 2014; Tracy et al. 2008), although several studies mention a link between sporulation and solvent production (Dürre 2014; Jones and Woods 1986; Long et al. 1984b; Rogers and Palosaari 1987; Schuster et al. 1998). Moreover, both cellular events can be lost simultaneously in a phenomenon named “strain degeneration,” which is observed after repeated batch fermentation or continuous fermentation (Clark et al. 1989; Cornillot et al. 1997; George et al. 1983; Jiao et al. 2016; Kashket and Cao 1995; Kosaka et al. 2007; Lv et al. 2016). Genomic and transcriptomic studies have been done on degenerated strains to unravel the mechanism of degeneration and find ways to prevent it. In *C. acetobutylicum*, it has been linked to the loss of the pSol megaplasmid, which contains genes encoding enzymes crucial for solvent production, an σ^X^ alternative sigma factor involved in sporulation (Behrens et al. 2000; Schuster et al. 2001) and SpoVD, a protein involved in the engulfment in *Bacillus* (Cornillot et al. 1997; Kashket and Cao 1995). In *C. beijerinckii* and *C. saccharoperbutylacetonicum*, which do not harbor the same type of megaplasmid, strain degeneration seems to be caused by mutations. Even though no consensus on the location, number, or type of mutations causing degeneration exists, the cell physiology of these degenerated strains is similar. Changes in medium composition were reported to prevent (Woo et al. 2018) or reestablish the solvent and sporulation ability, but the molecular mechanism is still unknown (Lv et al. 2016).

Nevertheless, common regulators of sporulation and solventogenesis have been identified. The study of cellular signaling pathways proved a close link between sporulation and solventogenesis initiation. The RRNPP quorum sensing systems seem to promote both cellular events. Several regulators enabling the transition from exponential phase to stationary phase are crucial to both cellular events. The disruption of general regulators such as CcpA and σ^L^ impaired both phenomena (Table 2). As for the role of Spo0A, it seems to differ depending on the species. In contrast to the phenotype described in *C. acetobutylicum* and *C. beijerinckii* (see section on the initiation of sporulation), the expression of *spo0A* is not required for solvent formation in *C. pasteurianum* (Schwarz et al. 2017). Proteins involved in Spo0A activation or inhibition, such as AbrB, orphan kinases, and σ^K^, contribute to the regulation of sporulation and solvent production. Studies showed, nonetheless, that decoupling sporulation and solvent formation is possible. Several solvent-producing but asporogenous mutants (Table 2) and recombinant strains have been isolated and engineered (Jones and Keis 1995; Li and He 2016).

### Spores in industrial processes

Sporulation in solventogenic clostridia is considered a drawback for several reasons. Once sporulation is initiated, cell growth stops, and the cell’s energy is used to generate metabolically inactive cells (Patakova et al. 2013). These events are undesirable in industrial settings as they negatively impact solvent productivity and cause cell wash-out in a continuous process (Li et al. 2020b; Papoutsakis 2008; Vees et al. 2020). Therefore, various attempts were made, like random mutagenesis, inactivation of early-stage sporulation proteins, and engineering of degenerated strain (Li et al. 2020b) to obtain asporogenous solventogenic strains or to control sporulation. No reports of inducible sporulation were made in solventogenic clostridia but in *C. difficile* (Dembek et al. 2017)*.* Dembek et al. controlled sporulation by introducing an anhydrotetracycline sensitive promoter upstream from *spo0A*. Alternatively, modifications of the process can reduce the proportion of sporulating cells. For instance, in continuous culture, the dilution rate is controlled to keep the cells in the growing phase to reduce sporulation (Mutschlechner et al. 2000). Interestingly, continuous cultures can also select for asporogenous solvent-producing strains (Meinecke et al. 1984). Meinecke et al. isolated in continuous culture under phosphate limitation a stable asporogenous strain which produced acetone and butanol as main products. For instance, in continuous culture, the dilution rate is controlled to keep the cells in the growing phase to reduce sporulation (Mutschlechner et al. 2000).

In contrast, bacterial spores can have useful applications, e.g., the pharmaceutical and agroindustry (Ricca and Cutting 2003; Wolken et al. 2003; Zhang et al. 2020). Their high resistance to heat and radiation makes them excellent bio-dosimeters. *B. subtilis* spores, for instance, are used to test the UV disinfection performance during drinking water purification (Mamane-Gravetz and Linden 2004). *B. subtilis* spores are also known to be effective biopesticides. The use of spores for enzyme immobilization is actively explored (Ugwuodo and Nwagu 2020). Compared to other immobilization supports, spores are very cheap, and several studies have highlighted the positive effects of spore immobilization on enzyme activity, stability and recovery (Falahati-Pour et al. 2015; Hosseini-Abari et al. 2016; Peng et al. 2020; Song et al. 2019). *Clostridium* spores are already used in the food and feed industry in Japan and China. Indeed, *C. butyricum* spores have been used as probiotics for several years. In fact, a large number of acid-producing *Clostridium* species are found in the gut of healthy individuals. They metabolize nutrients that cannot be degraded by the host. Studies have also proven that *C. butyricum* can prevent antibiotic-associated diarrhea and prevent *C. difficile* infections (Cartman 2011; Guo et al. 2020). The use of clostridial spores in the pharmaceutical industry is in the pipeline. Spores of *C. novyi* and *C. sporogenes* are being investigated as potential carriers for enzymes involved in chemotherapy (Dürre 2014; Kubiak and Minton 2015; Theys and Lambin 2015).

Some research on the integration of sporulation in the bioprocess for butanol production has been done. Spores can be used for cell immobilization. Low biomass is one of the issues of continuous culture with clostridia, and cell immobilization may prevent wash-out at high dilution rates (Vees et al. 2020). Spore can be immobilized on porous carriers (Dolejš et al. 2014; Krouwel et al. 1983) and microencapsulation (Rathore et al. 2015) to prevent cell wash-out. Microencapsulated spores can even be reused several times, enabling the production of butanol at high yields.

Moreover, sporulation enables consistent conservation of the strain characteristics, protecting the strains from stressors and degeneration. Due to the oxygen sensitivity of vegetative cells and the appearance of degenerative changes after repeated subculturing, spores are usually preferred for strain storage (Jones and Woods 1986). According to documentations on the industrial ABE processes from the 1920s to the 1980s, strains were stored as spores in sterile soil or sand in commercial plants (Jones and Woods 1986; Jones 2001). In South Africa’s ABE commercial plant, multiple cycles of germination/sporulation were performed to increase solvent productivity (Jones 2001). More recently, Vrije and co-workers explored the possibility of integrating a heat treatment during the product recovery by gas stripping in repeated batch cultures (de Vrije et al. 2013). This treatment killed the remaining vegetative cells and triggered the germination of the spores present in the culture, allowing the fermentation to start again after removing the solvents without the need for a new inoculation of the culture. This procedure improved solvent recovery and prolonged strain stability.

## Conclusion and perspectives for future studies

Next to solventogenesis, sporulation is a major stationary phase event occurring during ABE fermentation. Changes in carbon sources, media components, and cell density were described as the primary triggers of sporulation. Until recently, studies were mainly conducted with first-generation substrates, but with advanced biofuel production in mind, more studies on the effect of second and third-generation feedstocks on sporulation need to be realized.

Despite being a major part of the cell growth cycle, knowledge on its regulation mechanism in solvent-producing clostridia is scarce. Sporulation in *Bacillus* and pathogenic clostridia is well described, but noteworthy differences in the regulatory network exist between the two genera (Galperin 2013) and even within the clostridial pathogens (Shen et al. 2019). The model established for those species might not be applicable to solventogenic clostridia. As highlighted in a previous review (Patakova et al. 2013), most research is done on *C. acetobutylicum* ATCC 824, but with the advances in genome sequencing and gene engineering, more strains have become genetically accessible. Recent studies in other solventogenic strains have revealed differences in the transcriptional regulation of sporulation. Hence, more research in different solventogenic clostridia is required to understand better the complex regulation of sporulation and its interaction with other cellular events.

Furthermore, investigating the reasons behind the low sporulation frequency in cultures of acetogenic clostridia could give hints on strategies to take to prevent sporulation during the ABE fermentation. Knowledge in sporulation might be applied in the design of fermentation processes at different levels, by tailoring the medium composition to reduce/increase sporulation as desired or by integrating clostridial spore in the pretreatment of the substrate by displaying hydrolases on the spore surface or for cell immobilization during a continuous process. A better knowledge of sporulation in solventogenic and acetogenic clostridia would contribute to an improvement of the ABE and gas fermentation processes for the production of fuels and chemicals from renewable resources as a step towards a more sustainable industry.

## References

[CR1] Abd-Alla MH, Zohri ANA, El-Enany AWE, Ali SM (2017). Conversion of food processing wastes to biofuel using clostridia. Anaerobe.

[CR2] Abrini J, Naveau H, Nyns E-J (1994). *Clostridium autoethanogenum*, sp. nov., an anaerobic bacterium that produces ethanol from carbon monoxide. Arch Microbiol.

[CR3] Aframian N, Eldar A (2020). A bacterial tower of babel: quorum-sensing signaling diversity and its evolution. Annu Rev Microbiol.

[CR4] Aklujkar M, Leang C, Shrestha PM, Shrestha M, Lovley DR (2017). Transcriptomic profiles of *Clostridium ljungdahlii* during lithotrophic growth with syngas or H_2_ and CO_2_ compared to organotrophic growth with fructose. Sci Rep.

[CR5] Al-Hinai MA, Jones SW, Papoutsakis ET (2014). σ^K^ of *Clostridium acetobutylicum* is the first known sporulation-specific sigma factor with two developmentally separated roles, one early and one late in sporulation. J Bacteriol.

[CR6] Al-Hinai MA, Jones SW, Papoutsakis ET (2015). The *Clostridium* sporulation programs: diversity and preservation of endospore differentiation. Microbiol Mol Biol Rev.

[CR7] Alsaker KV, Spitzer TR, Papoutsakis ET (2004). Transcriptional analysis of *spo0A* overexpression in *Clostridium acetobutylicum* and its effect on the cell’s response to butanol stress. J Bacteriol.

[CR8] Annous BA, Blaschek HP (1991) Isolation and characterization of *Clostridium acetobutylicum* mutants with enhanced amylolytic activity. Appl Environ Microbiol 57(9) 10.1128/aem.57.9.2544-2548.1991 PMC183617 1722664

[CR9] Antunes A, Martin-Verstraete I, Dupuy B (2011). CcpA-mediated repression of *Clostridium difficile* toxin gene expression. Mol Microbiol.

[CR10] Atmadjaja AN, Holby V, Harding AJ, Krabben P, Smith HK, Jenkinson ER (2019) CRISPR-Cas, a highly effective tool for genome editing in *Clostridium saccharoperbutylacetonicum* N1-4(HMT). FEMS Microbiol Lett 366. 10.1093/femsle/fnz059 10.1093/femsle/fnz059 PMC6491355 30874768

[CR11] Avrova NP, Zubko IK, Alexeyeva EG (1981). The products of fermentation and the activity of pectolytic enzymes in *Clostridium felsineum* strains differing in the rate of spore formation. Mikrobiologiya.

[CR12] Awang GM, Ingledew WM, Jones GA (1992). The effect of fermentable carbohydrate on sporulation and butanol production by *Clostridium acetobutylicum* P262. Appl Microbiol Biotechnol.

[CR13] Barák I, Muchová K, Labajová N (2019). Asymmetric cell division during *Bacillus subtilis* sporulation. Future Microbiol.

[CR14] Basu A, Xin F, Lim TK, Lin Q, Yang KL, He J (2017). Quantitative proteome profiles help reveal efficient xylose utilization mechanisms in solventogenic *Clostridium* sp. strain BOH3. Biotechnol Bioeng.

[CR15] Bayat H, Modarressi MH, Rahimpour A (2018). The conspicuity of CRISPR-Cpf1 system as a significant breakthrough in genome editing. Curr Microbiol.

[CR16] Behrens S, Meyer U, Schankin H, Lonetto MA, Fischer RJ, Bahl H (2000). Identification of two genes encoding putative new members of the ECF subfamily of eubacterial RNA polymerase sigma factors in *Clostridium acetobutylicum*. J Mol Microbiol Biotechnol.

[CR17] Berezina O, Zakharova N, Yarotsky C, Zverlov V (2012). Microbial producers of butanol. Appl Biochem Microbiol.

[CR18] Bi C, Jones SW, Hess DR, Tracy MBP, Papoutsakis ET (2011). SpoIIe is necessary for asymmetric division, sporulation, and expression of σ^F^, σ^E^, and σ^G^ but does not control solvent production in *Clostridium acetobutylicum* ATCC 824. J Bacteriol.

[CR19] Bischofs IB, Hug JA, Liu AW, Wolf DM, Arkin AP (2009). Complexity in bacterial cell-cell communication: quorum signal integration and subpopulation signaling in the *Bacillus subtilis* phosphorelay. Proc Natl Acad Sci.

[CR20] Cañadas IC, Groothuis D, Zygouropoulou M, Rodrigues R, Minton NP (2019). RiboCas: a universal CRISPR-based editing tool for *Clostridium*. ACS Synth Biol.

[CR21] Cartman ST (2011). Time to consider *Clostridium* probiotics?. Future Microbiol.

[CR22] Chen Y, Zhou T, Liu D, Li A, Xu S, Liu Q, Li B, Ying H (2013). Production of butanol from glucose and xylose with immobilized cells of *Clostridium acetobutylicum*. Biotechnol Bioprocess Eng.

[CR23] Chen Y, Yang Y, Ji X, Zhao R, Li G, Gu Y, Shi A, Jiang W, Zhang Q (2020). The SCIFF-Derived ranthipeptides participate in quorum sensing in solventogenic clostridia. Biotechnol J.

[CR24] Cheng C, Bao T, Yang ST (2019). Engineering *Clostridium* for improved solvent production: recent progress and perspective. Appl Microbiol Biotechnol.

[CR25] Church BD (1959). Dependence of the heat resistance of bacterial endospores on their dipicolinic acid content. Nature.

[CR26] Clark SW, Bennett GN, Rudolph FB (1989). Isolation and characterization of mutants of *Clostridium acetobutylicum* ATCC 824 deficient in acetoacetyl-coenzyme a:acetate/butyrate:coenzyme a-transferase (EC 2.8.3.9) and in other solvent pathway enzymes. Appl Environ Microbiol.

[CR27] Collins M, Lawson P, Willems A, Cordoba JJ, Fernandez-Garayzabal J, Garcia P, Cai J, Hippe H, Farrow J (1994). The phylogeny of the genus *Clostridium*: proposal of five new genera and eleven new species combinations. Int J Syst Bacteriol.

[CR28] Cornillot E, Nair RV, Papoutsakis ET, Soucaille P (1997). The genes for butanol and acetone formation in *Clostridium acetobutylicum* ATCC 824 reside on a large plasmid whose loss leads to degeneration of the strain. J Bacteriol.

[CR29] Cruz-Morales P, Orellana CA, Moutafis G, Moonen G, Rincon G, Nielsen LK, Marcellin E, Bapteste E (2019). Revisiting the evolution and taxonomy of clostridia, a phylogenomic update. Genome Biol Evol.

[CR30] Davidson P, Eutsey R, Redler B, Hiller NL, Laub MT, Durand D (2018). Flexibility and constraint: evolutionary remodeling of the sporulation initiation pathway in *Firmicutes*. PLoS Genet.

[CR31] Dawes IW, Mandelstam J (1970). Sporulation of *Bacillus subtilis* in continuous culture. J Bacteriol.

[CR32] de Vrije T, Budde M, van der Wal H, Claassen PAM, López-Contreras AM (2013). “In situ” removal of isopropanol, butanol and ethanol from fermentation broth by gas stripping. Bioresour Technol.

[CR33] Decker AR, Ramamurthi KS (2017). Cell death pathway that monitors spore morphogenesis. Trends Microbiol.

[CR34] Dembek M, Willing SE, Hong HA, Hosseini S, Salgado PS, Cutting SM (2017). Inducible expression of *spo0A* as a universal tool for studying sporulation in *Clostridium difficile*. Front Microbiol.

[CR35] Desai RP, Papoutsakis ET (1999). Antisense RNA strategies for metabolic engineering of *Clostridium acetobutylicum*. Appl Environ Microbiol.

[CR36] Diallo M, Simons AD, van der Wal H, Collas F, Houweling-Tan B, Kengen SWM, López-Contreras AM (2018) L-Rhamnose metabolism in *Clostridium beijerinckii* strain DSM 6423. Appl Environ Microbiol 85(5). 10.1128/AEM.02656-18 10.1128/AEM.02656-18 PMC6384099 30578270

[CR37] Diallo M, Hocq R, Collas F, Chartier G, Wasels F, Wijaya HS, Werten MWT, Wolbert EJH, Kengen SWM, van der Oost J, Ferreira NL, López-Contreras AM (2020). Adaptation and application of a two-plasmid inducible CRISPR-Cas9 system in *Clostridium beijerinckii*. Methods.

[CR38] Diallo M, Kint N, Monot M, Collas F, Martin-Verstraete I, van der Oost J, Kengen SWM, López-Contreras AM (2020). Transcriptomic and phenotypic analysis of a *spoIIE* mutant in *Clostridium beijerinckii*. Front Microbiol.

[CR39] Diender M, Parera Olm I, Gelderloos M, Koehorst JJ, Schaap PJ, Stams AJM, Sousa DZ (2019). Metabolic shift induced by synthetic co-cultivation promotes high yield of chain elongated acids from syngas. Sci Rep.

[CR40] Dolejš I, Krasňan V, Stloukal R, Rosenberg M, Rebroš M (2014). Butanol production by immobilised *Clostridium acetobutylicum* in repeated batch, fed-batch, and continuous modes of fermentation. Bioresour Technol.

[CR41] Dürre P (2005) Handbook on clostridia. In: Duerre P (ed) Handbook on Clostridia. CRC Press. 10.1201/9780203489819

[CR42] Dürre P (2014) Physiology and sporulation in *Clostridium*. In The Bacterial Spore: from Molecules to Systems (Vol. 2, Issue 4, pp. 315–329). American Society of Microbiology. 10.1128/microbiolspec.TBS-0010-2012 10.1128/microbiolspec.TBS-0010-2012 26104199

[CR43] Edwards AN, Krall EG, McBride SM (2020) Strain-dependent RstA regulation of *Clostridioides difficile* toxin production and sporulation. J Bacteriol 202(2). 10.1128/JB.00586-19 10.1128/JB.00586-19 PMC6941533 31659010

[CR44] Ehsaan M, Kuit W, Zhang Y, Cartman ST, Heap JT, Winzer K, Minton NP (2016). Mutant generation by allelic exchange and genome resequencing of the biobutanol organism *Clostridium acetobutylicum* ATCC 824. Biotechnol Biofuels.

[CR45] Ezeji T, Qureshi N, Blaschek HP (2007). Butanol production from agricultural residues: Impact of degradation products on *Clostridium beijerinckii* growth and butanol fermentation. Biotechnol Bioeng.

[CR46] Falahati-Pour SK, Lotfi AS, Ahmadian G, Baghizadeh A (2015). Covalent immobilization of recombinant organophosphorus hydrolase on spores of *Bacillus subtilis*. J Appl Microbiol.

[CR47] Feng J, Zong W, Wang P, Zhang ZT, Gu Y, Dougherty M, Borovok I, Wang Y (2020) RRNPP-Type quorum-sensing systems regulate solvent formation, sporulation and cell motility in *Clostridium**saccharoperbutylacetonicum*. Biotechnol Biofuels 13(1):84. 10.1186/s13068-020-01723-x 10.1186/s13068-020-01723-x PMC7206700 32411297

[CR48] Fimlaid KA, Shen A (2015). Diverse mechanisms regulate sporulation sigma factor activity in the *Firmicutes*. Curr Opin Microbiol.

[CR49] Forsberg CW (1987). Production of 1,3-Propanediol from glycerol by *Clostridium acetobutylicum* and other *Clostridium* species. Appl Environ Microbiol.

[CR50] Foulquier C, Huang C-N, Nguyen N-P-T, Thiel A, Wilding-Steel T, Soula J, Yoo M, Ehrenreich A, Meynial-Salles I, Liebl W, Soucaille P (2019). An efficient method for markerless mutant generation by allelic exchange in *Clostridium acetobutylicum* and *Clostridium saccharobutylicum* using suicide vectors. Biotechnol Biofuels.

[CR51] Freedman JC, Li J, Mi E, McClane BA (2019). Identification of an important orphan histidine kinase for the initiation of sporulation and enterotoxin production by *Clostridium perfringens* Type F strain SM101. MBio.

[CR52] Fujita M, González-Pastor JE, Losick R (2005). High- and low-threshold genes in the Spo0A regulon of *Bacillus subtilis*. J Bacteriol.

[CR53] Gallazzi A, Branska B, Marinelli F, Patakova P (2015). Continuous production of n-butanol by *Clostridium pasteurianum* DSM 525 using suspended and surface-immobilized cells. J Biotechnol.

[CR54] Galperin MY (2013) Genome diversity of spore-forming *Firmicutes*. Microbiol Spectr 1(2). 10.1128/microbiolspectrum.TBS-0015-2012 10.1128/microbiolspectrum.TBS-0015-2012 PMC4306282 26184964

[CR55] Galperin MY, Mekhedov SL, Puigbo P, Smirnov S, Wolf YI, Rigden DJ (2012). Genomic determinants of sporulation in Bacilli and Clostridia : towards the minimal set of sporulation-specific genes. Environ Microbiol.

[CR56] George HA, Johnson JL, Moore WE, Holdeman LV, Chen JS (1983). Acetone, isopropanol, and butanol production by *Clostridium beijerinckii* (syn. *Clostridium butylicum*) and *Clostridium aurantibutyricum*. Appl Environ Microbiol.

[CR57] Gong F, Bao G, Zhao C, Zhang Y, Li Y, Dong H (2016). Fermentation and genomic analysis of acetone-uncoupled butanol production by *Clostridium tetanomorphum*. Appl Microbiol Biotechnol.

[CR58] Green EM (2011). Fermentative production of butanol—the industrial perspective. Curr Opin Biotechnol.

[CR59] Green EM, Boynton ZL, Harris LM, Rudolph FB, Papoutsakis ET, Bennett GN (1996). Genetic manipulation of acid formation pathways by gene inactivation in *Clostridium acetobutylicum* ATCC 824. Microbiology.

[CR60] Grimmler C, Janssen H, Krauße D, Fischer R-JJ, Bahl H, Dürre P, Liebl W, Ehrenreich A (2011). Genome-wide gene expression analysis of the switch between acidogenesis and solventogenesis in continuous cultures of *Clostridium acetobutylicum*. J Mol Microbiol Biotechnol.

[CR61] Grossman AD, Losick R (1988). Extracellular control of spore formation in *Bacillus subtilis*. Proc Natl Acad Sci U S A.

[CR62] Guo P, Zhang K, Ma X, He P (2020). *Clostridium* species as probiotics: potentials and challenges. J Anim Sci Biotechnol.

[CR63] Gupta RS, Gao B (2009). Phylogenomic analyses of clostridia and identification of novel protein signatures that are specific to the genus *Clostridium* sensu stricto (cluster I). Int J Syst Evol Microbiol.

[CR64] Harris LM, Desai RP, Welker NE, Papoutsakis ET (2000). Characterization of recombinant strains of the *Clostridium acetobutylicum* butyrate kinase inactivation mutant: need for new phenomenological models for solventogenesis and butanol inhibition?. Biotechnol Bioeng.

[CR65] Harris LM, Welker NE, Papoutsakis ET (2002). Northern, morphological, and fermentation analysis of *spo0A* inactivation and overexpression in *Clostridium acetobutylicum* ATCC 824. J Bacteriol.

[CR66] Hecker M, Völker U (2001) General stress response of *Bacillus subtilis* and other bacteria. In Advances in Microbial Physiology (Vol. 44, pp. 35–91). Academic Press. 10.1016/S0065-2911(01)44011-2 10.1016/s0065-2911(01)44011-2 11407115

[CR67] Heluane H, Dagher MRE, Bruno-Bárcena JM, Evans MR, Dagher SF, Bruno-Bárcena JM (2011). Meta-analysis and functional validation of nutritional requirements of solventogenic clostridia growing under butanol stress conditions and coutilization of D-glucose and D-xylose. Appl Environ Microbiol.

[CR68] Herman NA, Kim SJ, Li JS, Cai W, Koshino H, Zhang W (2017). The industrial anaerobe *Clostridium acetobutylicum* uses polyketides to regulate cellular differentiation. Nat Commun.

[CR69] Hocq R, Bouilloux-Lafont M, Lopes Ferreira N, Wasels F (2019). σ^54^ (σ^L^) plays a central role in carbon metabolism in the industrially relevant *Clostridium beijerinckii*. Sci Rep.

[CR70] Hosseini-Abari A, Kim B-G, Lee S-H, Emtiazi G, Kim W, Kim J-H (2016). Surface display of bacterial tyrosinase on spores of *Bacillus subtilis* using CotE as an anchor protein. J Basic Microbiol.

[CR71] Hu S, Zheng H, Gu Y, Zhao J, Zhang W, Yang Y, Wang S, Zhao G, Yang S, Jiang W (2011). Comparative genomic and transcriptomic analysis revealed genetic characteristics related to solvent formation and xylose utilization in *Clostridium acetobutylicum* EA 2018. BMC Genomics.

[CR72] Huang SS, Chen D, Pelczar PL, Vepachedu VR, Setlow P, Li YQ (2007). Levels of Ca^2+^-dipicolinic acid in individual *Bacillus* spores determined using microfluidic Raman tweezers. J Bacteriol.

[CR73] Huang H, Chai C, Yang S, Jiang W, Gu Y (2019). Phage serine integrase-mediated genome engineering for efficient expression of chemical biosynthetic pathway in gas-fermenting *Clostridium ljungdahlii*. Metab Eng.

[CR74] Huhnke RL, Lewis RS, Tanner RS (2008) Isolation and characterization of novel clostridial species (Patent No. US7704723 B2). In Patent (US7704723 B2). U.S. Patent and Trademark Office. https://shareok.org/handle/11244/15409

[CR75] Jabbari S, Steiner E, Heap JT, Winzer K, Minton NP, King JR (2013). The putative influence of the *agr* operon upon survival mechanisms used by *Clostridium acetobutylicum*. Math Biosci.

[CR76] Jain MK, Beacom D, Datta R, Lansing E (1994) Mutant strain of *C. acetobutylicum* and process for making butanol (Patent No. US5192673A). In *Biotechnology Advances* (No. US5192673A). U.S. Patent and Trademark Office. 10.1016/0734-9750(94)90895-8

[CR77] Jamroskovic J, Chromikova Z, List C, Bartova B, Barák I, Bernier-Latmani R (2016). Variability in DPA and calcium content in the spores of *Clostridium* species. Front Microbiol.

[CR78] Jiao S, Zhang Y, Wan C, Lv J, Du R, Zhang R, Han B (2016). Transcriptional analysis of degenerate strain *Clostridium beijerinckii* DG-8052 reveals a pleiotropic response to CaCO_3-_ associated recovery of solvent production. Sci Rep.

[CR79] Johnson EA (2019) Clostridia. Encycl Microbiol 690–695. 10.1016/B978-0-12-801238-3.98821-4

[CR80] Jones DT (2001) Applied acetone-butanol fermentation. In H Bahl & P Dürre (eds) *Clostridia* (Vol. 2, Issue 1, pp. 125–168). Wiley-VCH Verlag GmbH. 10.1002/3527600108.ch5

[CR81] Jones DT, Keis S (1995). Origins and relationships of industrial solvent-producing clostridial strains. FEMS Microbiol. Rev..

[CR82] Jones DT, Woods DR (1986). Acetone-butanol fermentation revisited. Microbiol Rev.

[CR83] Jones SW, Paredes CJ, Tracy B, Cheng N, Sillers R, Senger RS, Papoutsakis ET (2008). The transcriptional program underlying the physiology of clostridial sporulation. Genome Biol.

[CR84] Jones SW, Tracy BP, Gaida SM, Papoutsakis ET (2011) Inactivation of σ^F^ in *Clostridium acetobutylicum* ATCC 824 blocks sporulation prior to asymmetric division and abolishes σ^E^ and σ^G^ protein expression but does not block solvent formation. J Bacteriol 193(10):2429–2440. 10.1128/JB.00088-11 10.1128/JB.00088-11 PMC3133174 21421765

[CR85] Joseph RC, Kim NM, Sandoval NR (2018). Recent developments of the synthetic biology toolkit for *Clostridium*. Front Microbiol.

[CR86] Kashket ER, Cao Z-Y (1995). Clostridial strain degeneration. FEMS Microbiol. Rev..

[CR87] Kay D, Warren SC (1968). Sporulation in *Bacillus subtilis*. Morphological changes. Biochem J.

[CR88] Keis S, Bennett CF, Ward VK, Jones DT (1995). Taxonomy and phylogeny of industrial solvent-producing clostridia. Int J Syst Bacteriol.

[CR89] Keis S, Shaheen R, Jones DT (2001). Emended descriptions of *Clostridium acetobutylicum* and *Clostridium beijerinckii*, and descriptions of *Clostridium saccharoperbutylacetonicum* sp nov and *Clostridium saccharobutylicum* sp nov. Int J Syst Evol Microbiol.

[CR90] Kihm DJ, Hutton MT, Hanlin JH, Johnson EA (1988). Zinc stimulates sporulation in *Clostridium botulinum* 113B. Curr Microbiol.

[CR91] Kiyoshi K, Kawashima S, Nobuki K, Kadokura T, Nakazato A, Suzuki KI, Nakayama S (2017). Adenine addition restores cell viability and butanol production in *Clostridium saccharoperbutylacetonicum* N1-4 (ATCC 13564) cultivated at 37°C. Appl Environ Microbiol.

[CR92] Knaysi G (1948). The endospore of bacteria. Bacteriol Rev.

[CR93] Kolek J, Diallo M, Vasylkivska M, Branska B, Sedlar K, López-Contreras AM, Patakova P (2017). Comparison of expression of key sporulation, solventogenic and acetogenic genes in *C. beijerinckii* NRRL B-598 and its mutant strain overexpressing *spo0A*. Appl Microbiol Biotechnol.

[CR94] Kosaka T, Nakayama S, Nakaya K, Yoshino S, Furukawa K (2007). Characterization of the sol operon in butanol-hyperproducing *Clostridium saccharoperbutylacetonicum* strain N1-4 and its degeneration mechanism. Biosci Biotechnol Biochem.

[CR95] Kosono S, Tamura M, Suzuki S, Kawamura Y, Yoshida A, Nishiyama M, Yoshida M (2015). Changes in the acetylome and succinylome of *Bacillus subtilis* in response to carbon source. PLoS One.

[CR96] Kotte A-K, Severn O, Bean Z, Schwarz K, Minton NP, Winzer K (2020). RRNPP-type quorum sensing affects solvent formation and sporulation in *Clostridium acetobutylicum*. Microbiology.

[CR97] Król E, De Sousa BA, Kopacz M, Scheffers DJ (2017). Metal-dependent SpoIIE oligomerization stabilizes FtsZ during asymmetric division in *Bacillus subtilis*. PLoS One.

[CR98] Krouwel PG, Groot WJ, Kossen NWF, van der Laan WFM (1983). Continuous isopropanol-butanol-ethanol fermentation by immobilized *Clostridium beijerinckii* cells in a packed bed fermenter. Enzym Microb Technol.

[CR99] Kubiak AM, Minton NP (2015). The potential of clostridial spores as therapeutic delivery vehicles in tumour therapy. Res Microbiol.

[CR100] Kuit W (2013) Metabolic engineering of acid formation in *Clostridium acetobutylicum* Wouter Kuit [Wageningen University]. http://library.wur.nl/WebQuery/wurpubs/fulltext/241021

[CR101] Kuit W, Minton NP, López-Contreras AM, Eggink G (2012) Disruption of the acetate kinase (*ack*) gene of *Clostridium acetobutylicum* results in delayed acetate production. Appl Microbiol Biotechnol 94(3):729–741. 10.1007/s00253-011-3848-4 10.1007/s00253-011-3848-4 PMC3315644 22249720

[CR102] LanzaTech (2019) LanzaTech | Capturing carbon. Fueling growth. LanzaTech. https://www.lanzatech.com/

[CR103] Lawson PA, Rainey FA (2016) Proposal to restrict the genus *Clostridium* Prazmowski to *Clostridium butyricum* and related species. Int J Syst Evol Microbiol 66(2):1009–1016. 10.1099/ijsem.0.000824 10.1099/ijsem.0.000824 26643615

[CR104] Lee KY, Juang TC, Lee KC (1978). Effect of metal ions on growth and sporulation of *Clostridium perfringens* in a synthetic medium. Chin J Microbiol Immunol.

[CR105] Lee AJ, Byun BY, Kang D-H, Tang J, Kim Y-W, Hwang H-J, Mah J-H (2011) The ability of zinc to inhibit the sporulation and viability of *Clostridium**sporogenes* and growth of other bacteria. Int J Food Sci Technol 46(7):1494–1501. 10.1111/j.1365-2621.2011.02644.x

[CR106] Lee S, Lee JH, Mitchell RJ (2015). Analysis of *Clostridium beijerinckii* NCIMB 8052’s transcriptional response to ferulic acid and its application to enhance the strain tolerance. Biotechnol Biofuels.

[CR107] Leggett MJ, Mcdonnell G, Denyer SP, Setlow P, Maillard JY (2012). Bacterial spore structures and their protective role in biocide resistance. J Appl Microbiol.

[CR108] Lenz CA, Vogel RF (2014). Effect of sporulation medium and its divalent cation content ontheheat and high pressure resistance of *Clostridium botulinum* typeE spores. Food Microbiol.

[CR109] Letzel A-C, Pidot SJ, Hertweck C (2013). A genomic approach to the cryptic secondary metabolome of the anaerobic world. Nat Prod Rep.

[CR110] Li T, He J (2016). Simultaneous saccharification and fermentation of hemicellulose to butanol by a non-sporulating *Clostridium* species. Bioresour Technol.

[CR111] Li J, Chen J, Vidal JE, McClane BA (2011). The Agr-like quorum-sensing system regulates sporulation and production of enterotoxin and beta2 toxin by *Clostridium perfringens* type a non-food-borne human gastrointestinal disease strain F5603. Infect Immun.

[CR112] Li Q, Chen J, Minton NP, Zhang Y, Wen Z, Liu J, Yang H, Zeng Z, Ren X, Yang J, Gu Y, Jiang W, Jiang Y, Yang S (2016). CRISPR-based genome editing and expression control systems in *Clostridium acetobutylicum* and *Clostridium beijerinckii*. Biotechnol J.

[CR113] Li Q, Seys FM, Minton NP, Yang J, Jiang Y, Jiang W, Yang S (2019). CRISPR-Cas9 D10A nickase-assisted base editing in solvent producer *Clostridium beijerinckii*. Biotechnol Bioeng.

[CR114] Li JS, Barber CC, Herman NA, Cai W, Zafrir E, Du Y, Zhu X, Skyrud W, Zhang W (2020). Investigation of secondary metabolism in the industrial butanol hyper-producer *Clostridium saccharoperbutylacetonicum* N1-4. J Ind Microbiol Biotechnol.

[CR115] Li S, Huang L, Ke C, Pang Z, Liu L (2020). Pathway dissection, regulation, engineering and application: lessons learned from biobutanol production by solventogenic clostridia. Biotechnol Biofuels.

[CR116] Liew F, Martin ME, Tappel RC, Heijstra BD, Mihalcea C, Köpke M (2016) Gas Fermentation—a flexible platform for commercial scale production of low-carbon-fuels and chemicals from waste and renewable feedstocks. Front Microbiol 7(694). 10.3389/fmicb.2016.00694 10.3389/fmicb.2016.00694 PMC4862988 27242719

[CR117] Liou JS-C, Balkwill DL, Drake GR, Tanner RS (2005). *Clostridium carboxidivorans* sp. nov., a solvent-producing *Clostridium* isolated from an agricultural settling lagoon, and reclassification of the acetogen *Clostridium scatologenes* strain SL1 as *Clostridium drakei* sp. nov. Int J Syst Evol Microbiol.

[CR118] List C, Hosseini Z, Lederballe Meibom K, Hatzimanikatis V, Bernier-Latmani R (2019). Impact of iron reduction on the metabolism of *Clostridium acetobutylicum*. Environ Microbiol.

[CR119] Liu Z, Qiao K, Tian L, Zhang Q, Liu Z-Y, Li F-L (2015). Spontaneous large-scale autolysis in *Clostridium acetobutylicum* contributes to generation of more spores. Front Microbiol.

[CR120] Liu H, Zhang J, Yuan J, Jiang X, Jiang L, Li Z, Yin Z, Du Y, Zhao G, Liu B, Huang D (2020). Gene coexpression network analysis reveals a novel metabolic mechanism of *Clostridium acetobutylicum* responding to phenolic inhibitors from lignocellulosic hydrolysates. Biotechnol Biofuels.

[CR121] Long S, Jones DT, Woods DR (1983). Sporulation of *Clostridium acetobutylicum* P262 in a Defined Medium. Appl Environ Microbiol.

[CR122] Long S, Jones D, Woods D (1984a) Initiation of solvent production, clostridial stage and endospore formation in *Clostridium acetobutylicum* P262. Appl Microbiol Biotechnol 20(4). 10.1007/BF00250635

[CR123] Long S, Jones DT, Woods DR (1984). The relationship between sporulation and solvent production in *Clostridium acetobutylicum* P262. Biotechnol Lett.

[CR124] Lund BM, Brocklehurst TF, Wyatt GM (1981). Characterization of strains of *Clostridium puniceum* sp.nov., a pink-pigmented, pectolytic bacterium. J Gen Microbiol.

[CR125] Lv J, Jiao S, Du R, Zhang R, Zhang Y, Han B (2016). Proteomic analysis to elucidate degeneration of *Clostridium beijerinckii* NCIMB 8052 and role of Ca^2+^ in strain recovery from degeneration. J Ind Microbiol Biotechnol.

[CR126] Macek B, Forchhammer K, Hardouin J, Weber-Ban E, Grangeasse C, Mijakovic I (2019). Protein post-translational modifications in bacteria. Nat Rev Microbiol.

[CR127] Mah JH, Kang DH, Tang J (2008). Effects of minerals on sporulation and heat resistance of *Clostridium sporogenes*. Int J Food Microbiol.

[CR128] Malaviya A, Jang YS, Lee SY (2012). Continuous butanol production with reduced byproducts formation from glycerol by a hyper producing mutant of *Clostridium pasteurianum*. Appl Microbiol Biotechnol.

[CR129] Mamane-Gravetz H, Linden KG (2004). UV disinfection of indigenous aerobic spores: Implications for UV reactor validation in unfiltered waters. Water Res.

[CR130] Martínez-Lumbreras S, Alfano C, Evans NJ, Collins KM, Flanagan KA, Atkinson RA, Krysztofinska EM, Vydyanath A, Jackter J, Fixon-Owoo S, Camp AH, Isaacson RL (2018). Structural and functional insights into *Bacillus subtilis* sigma factor inhibitor, CsfB. Structure.

[CR131] Mate de Gerando H, Wasels F, Bisson A, Clement B, Bidard F, & Jourdier E. (2018). Genome sequence of the natural isopropanol producer *Clostridium beijerinckii* DSM 6423. https://www.ebi.ac.uk/ena/browser/view/PRJEB11626 10.1186/s12864-018-4636-7 PMC5894183 29636009

[CR132] Máté de Gérando H, Wasels F, Bisson A, Clement B, Bidard F, Jourdier E, López-Contreras AM, Lopes Ferreira N (2018). Genome and transcriptome of the natural isopropanol producer *Clostridium beijerinckii* DSM 6423. BMC Genomics.

[CR133] Meinecke B, Bahl H, Gottschalk G (1984). Selection of an asporogenous strain of *Clostridium acetobutylicum* in continuous culture under phosphate limitation. Appl Environ Microbiol.

[CR134] Mukherjee M, Sarkar P, Goswami G, Das D (2019). Regulation of butanol biosynthesis in *Clostridium acetobutylicum* ATCC 824 under the influence of zinc supplementation and magnesium starvation. Enzym Microb Technol.

[CR135] Mutschlechner O, Swoboda H, Gapes JR (2000). Continuous two-stage ABE-fermentation using *Clostridium beijerinckii* NRRL B592 operating with a growth rate in the first stage vessel close to its maximal value. J Mol Microbiol Biotechnol.

[CR136] Narula J, Devi SN, Fujita M, Igoshin OA (2012). Ultrasensitivity of the *Bacillus subtilis* sporulation decision. Proc Natl Acad Sci.

[CR137] Nimbalkar PR, Khedkar MA, Parulekar RS, Chandgude VK, Sonawane KD, Chavan PV, Bankar SB (2018). Role of trace elements as cofactor: an efficient strategy toward enhanced biobutanol production. ACS Sustain Chem Eng.

[CR138] Nimbalkar PR, Khedkar MA, Chavan PV, Bankar SB (2019). Enhanced biobutanol production in folic acid-induced medium by using *Clostridium acetobutylicum* NRRL B-527. ACS Omega.

[CR139] Paidhungat M, Setlow B, Driks A, Setlow P (2000). Characterization of spores of *Bacillus subtilis* which lack dipicolinic acid. J Bacteriol.

[CR140] Papoutsakis ET (2008). Engineering solventogenic clostridia. Curr Opin Biotechnol.

[CR141] Paredes CJ, Alsaker KV, Papoutsakis ET (2005). A comparative genomic view of clostridial sporulation and physiology. Nat Rev Microbiol.

[CR142] Paredes-Sabja D, Setlow B, Setlow P, Sarker MR (2008). Characterization of *Clostridium perfringens* spores that lack SpoVA proteins and dipicolinic acid. J Bacteriol.

[CR143] Paredes-Sabja D, Shen A, Sorg JA (2014). *Clostridium difficile* spore biology: sporulation, germination, and spore structural proteins. Trends Microbiol.

[CR144] Patakova P, Linhova M, Rychtera M, Paulova L, Melzoch K (2013). Novel and neglected issues of acetone-butanol-ethanol (ABE) fermentation by clostridia: *Clostridium* metabolic diversity, tools for process mapping and continuous fermentation systems. Biotechnol Adv.

[CR145] Patakova P, Linhova M, Vykydalova P, Branska B, Rychtera M, Melzoch K (2014). Use of fluorescent staining and flow cytometry for monitoring physiological changes in solventogenic clostridia. Anaerobe.

[CR146] Patakova P, Branska B, Sedlar K, Vasylkivska M, Jureckova K, Kolek J, Koscova P, Provaznik I (2019). Acidogenesis, solventogenesis, metabolic stress response and life cycle changes in *Clostridium beijerinckii* NRRL B-598 at the transcriptomic level. Sci Rep.

[CR147] Paul C, Filippidou S, Jamil I, Kooli W, House GL, Estoppey A, Hayoz M, Junier T, Palmieri F, Wunderlin T, Lehmann A, Bindschedler S, Vennemann T, Chain PSG, Junier P (2019). Bacterial spores, from ecology to biotechnology. Adv Appl Microbiol.

[CR148] Peng F, Zheng B, Zhang Y, Faheem A, Chai Y, Jiang T, Chen X, Hu Y (2020). Biocatalytic oxidation of aromatic compounds by spore-based system. ACS Sustain Chem Eng.

[CR149] Philips J, Rabaey K, Lovley DR, Vargas M (2017). Biofilm formation by *Clostridium ljungdahlii* is induced by sodium chloride stress: experimental evaluation and transcriptome analysis. PLoS One.

[CR150] Piggot PJ, Hilbert DW (2004). Sporulation of *Bacillus subtilis*. Curr Opin Microbiol.

[CR151] Poehlein A, Solano JDM, Flitsch SK, Krabben P, Winzer K, Reid SJ, Jones DT, Green E, Minton NP, Daniel R, Dürre P (2017). Microbial solvent formation revisited by comparative genome analysis. Biotechnol Biofuels.

[CR152] Prazmowski A (1880) Untersuchungen über die Entwickelungsgeschichte und Fermentwirkung einiger Bacterien-Arten. Voigt.

[CR153] Qureshi N, Blaschek HP (2001). Recent advances in ABE fermentation: hyper-butanol producing *Clostridium beijerinckii* BA101. J Ind Microbiol Biotechnol.

[CR154] Raedts J, Siemerink MAJ, Levisson M, van der Oost J, Kengen SWM (2014). Molecular characterization of an NADPH-dependent acetoin reductase/2,3-butanediol dehydrogenase from *Clostridium beijerinckii* NCIMB 8052. Appl Environ Microbiol.

[CR155] Rathore S, Wan Sia Heng P, Chan LW (2015). Microencapsulation of *Clostridium acetobutylicum* ATCC 824 spores in gellan gum microspheres for the production of biobutanol. J Microencapsul.

[CR156] Ravagnani A, Jennert KCB, Steiner E, Grünberg R, Jefferies JR, Wilkinson SR, Young DI, Tidswell EC, Brown DP, Youngman P, Gareth Morris J, Young M (2000). Spo0A directly controls the switch from acid to solvent production in solvent-forming clostridia. Mol Microbiol.

[CR157] Reeve BWP, Reid SJ (2016). Glutamate and histidine improve both solvent yields and the acid tolerance response of *Clostridium beijerinckii* NCP 260. J Appl Microbiol.

[CR158] Ren C, Gu Y, Wu Y, Zhang W, Yang C, Yang S, Jiang W (2012). Pleiotropic functions of catabolite control protein CcpA in Butanol-producing *Clostridium acetobutylicum*. BMC Genomics.

[CR159] Ricca E, Cutting SM (2003). Emerging applications of bacterial spores in nanobiotechnology. J Nanobiotechnol.

[CR160] Rogers P, Palosaari N (1987). *Clostridium acetobutylicum* mutants that produce butyraldehyde and altered quantities of solvents. Appl Environ Microbiol.

[CR161] Sandoval NR, Venkataramanan KP, Groth TS, Papoutsakis ET (2015). Whole-genome sequence of an evolved *Clostridium pasteurianum* strain reveals Spo0A deficiency responsible for increased butanol production and superior growth. Biotechnol Biofuels.

[CR162] Sandoval-Espinola WJ, Makwana ST, Chinn MS, Thon MR, Azcárate-Peril MA, Bruno-Bárcena JM (2013). Comparative phenotypic analysis and genome sequence of *Clostridium beijerinckii* SA-1, an offspring of NCIMB 8052. Microbiology.

[CR163] Sauer M (2016) Industrial production of acetone and butanol by fermentation-100 years later. FEMS Microbiol Lett 363(13). 10.1093/femsle/fnw134 10.1093/femsle/fnw134 PMC4894279 27199350

[CR164] Sauer U, Santangelo JD, Treuner A, Buchholz M, Dürre P (1995). Sigma factor and sporulation genes in *Clostridium*. FEMS Microbiol Rev.

[CR165] Schmeisser F, Brannigan JA, Lewis RJ, Wilkinson AJ, Youngman P, BarÃ¡k I (2000). A new mutation in *spo0A* with intragenic suppressors in the effector domain. FEMS Microbiol Lett.

[CR166] Schuster KC, Van Den Heuvel R, Gutierrez NA, Maddox IS (1998). Development of markers for product formation and cell cycle in batch cultivation of *Clostridium acetobutylicum* ATCC 824. Appl Microbiol Biotechnol.

[CR167] Schuster K, Goodacre R, Gapes J, Young M (2001). Degeneration of solventogenic *Clostridium* strains monitored by Fourier transform infrared spectroscopy of bacterial cells. J Ind Microbiol Biotechnol.

[CR168] Schwarz KM, Kuit W, Grimmler C, Ehrenreich A, Kengen SWM (2012). A transcriptional study of acidogenic chemostat cells of *Clostridium acetobutylicum* — cellular behavior in adaptation to n-butanol. J Biotechnol.

[CR169] Schwarz KM, Grosse-Honebrink A, Derecka K, Rotta C, Zhang Y, Minton NP (2017). Towards improved butanol production through targeted genetic modification of *Clostridium pasteurianum*. Metab Eng.

[CR170] Scotcher MC, Bennett GN (2005). SpoIIE regulates sporulation but does not directly affect solventogenesis in *Clostridium acetobutylicum* ATCC 824. J Bacteriol.

[CR171] Scotcher MC, Rudolph FB, Bennett GN (2005). Expression of *abrB310* and *sinR*, and effects of decreased *abrB310* expression on the transition from acidogenesis to solventogenesis, in *Clostridium acetobutylicum* ATCC 824. Appl Environ Microbiol.

[CR172] Sedlar K, Koscova P, Vasylkivska M, Branska B, Kolek J, Kupkova K, Patakova P, Provaznik I (2018). Transcription profiling of butanol producer *Clostridium beijerinckii* NRRL B-598 using RNA-Seq. BMC Genomics.

[CR173] Sedlar K, Kolek J, Gruber M, Jureckova K, Branska B, Csaba G, Vasylkivska M, Zimmer R, Patakova P, Provaznik I (2019). A transcriptional response of *Clostridium beijerinckii* NRRL B-598 to a butanol shock. Biotechnol Biofuels.

[CR174] Sedlar K, Vasylkivska M, Musilova J, Branska B, Provaznik I, Patakova P (2021) Phenotypic and genomic analysis of isopropanol and 1,3-propanediol producer *Clostridium diolis *DSM 15410. Genomics 113(1):1109–1119. 10.1016/j.ygeno.2020.11.007 10.1016/j.ygeno.2020.11.007 33166602

[CR175] Seo SO, Janssen H, Magis A, Wang Y, Lu T, Price ND, Jin YS, Blaschek HP (2017). Genomic, transcriptional, and phenotypic analysis of the glucose derepressed *Clostridium beijerinckii* mutant exhibiting acid crash phenotype. Biotechnol J.

[CR176] Seo SO, Wang Y, Lu T, Jin YS, Blaschek HP (2017). Characterization of a *Clostridium beijerinckii spo0A* mutant and its application for butyl butyrate production. Biotechnol Bioeng.

[CR177] Seo SO, Lu T, Jin Y-S, Blaschek HP (2021). A comparative phenotypic and genomic analysis of *Clostridium beijerinckii* mutant with enhanced solvent production. J Biotechnol.

[CR178] Serrano M, Crawshaw AD, Dembek M, Monteiro JM, Pereira FC, Pinho MG, Fairweather NF, Salgado PS, Henriques AO (2016). The SpoIIQ-SpoIIIAH complex of *Clostridium difficile* controls forespore engulfment and late stages of gene expression and spore morphogenesis. Mol Microbiol.

[CR179] Seys FM, Rowe P, Bolt EL, Humphreys CM, Minton NP (2020). A gold standard, CRISPR/Cas9-based complementation strategy reliant on 24 nucleotide bookmark sequences. Genes (Basel).

[CR180] Shaheen R, Shirley M, Jones DT (2000). Comparative fermentation studies of industrial strains belonging to four species of solvent-producing clostridia. J Mol Microbiol Biotechnol.

[CR181] Shen A, Edwards AN, Sarker MR, Paredes-Sabja D (2019) Sporulation and germination in clostridial pathogens. Microbiol Spectr 7(6). 10.1128/microbiolspec.gpp3-0017-2018 10.1128/microbiolspec.gpp3-0017-2018 PMC6927485 31858953

[CR182] Shi Z, Blaschek HP (2008). Transcriptional analysis of *Clostridium beijerinckii* NCIMB 8052 and the hyper-butanol-producing mutant BA101 during the shift from acidogenesis to solventogenesis. Appl Environ Microbiol.

[CR183] Sjolander NO, Langlykke AF, Peterson WH (1938). Butyl alcohol fermentation of wood sugar. Ind Eng Chem.

[CR184] Song T, Wang F, Xiong S, Jiang H (2019). Surface display of organophosphorus-degrading enzymes on the recombinant spore of *Bacillus subtilis*. Biochem Biophys Res Commun.

[CR185] Steiner E, Dago AE, Young DI, Heap JT, Minton NP, Hoch JA, Young M (2011). Multiple orphan histidine kinases interact directly with Spo0A to control the initiation of endospore formation in *Clostridium acetobutylicum*. Mol Microbiol.

[CR186] Steiner E, Scott J, Minton NP, Winzer K (2012). An agr quorum sensing system that regulates granulose formation and sporulation in *Clostridium acetobutylicum*. Appl Environ Microbiol.

[CR187] Sullivan L, Bennett GN (2006). Proteome analysis and comparison of *Clostridium acetobutylicum* ATCC 824 and Spo0A strain variants. J Ind Microbiol Biotechnol.

[CR188] Tashiro Y, Yoshida T, Noguchi T, Sonomoto K (2013). Recent advances and future prospects for increased butanol production by acetone-butanol-ethanol fermentation. Eng Life Sci.

[CR189] Terracciano JS, Kashket ER (1986). Intracellular conditions required for initiation of solvent production by *Clostridium acetobutylicum*. Appl Environ Microbiol.

[CR190] Theys J, Lambin P (2015). *Clostridium* to treat cancer: dream or reality?. Ann Transl Med.

[CR191] Thorn GJ, King JR, Jabbari S (2013). pH-induced gene regulation of solvent production by *Clostridium acetobutylicum* in continuous culture: parameter estimation and sporulation modelling. Math Biosci.

[CR192] Tokuyasu K, Yamada E (1959). Fine structure of *Bacillus subtilis*. J Biophys Biochem Cytol.

[CR193] Tomas CA, Beamish J, Papoutsakis ET (2004). Transcriptional analysis of butanol stress and tolerance in *Clostridium acetobutylicum*. J Bacteriol.

[CR194] Traag BA, Pugliese A, Eisen JA, Losick R (2013). Gene conservation among endospore-forming bacteria reveals additional sporulation genes in *Bacillus subtilis*. J Bacteriol.

[CR195] Tracy BP, Gaida SM, Papoutsakis ET (2008). Development and application of flow-cytometric techniques for analyzing and sorting endospore-forming clostridia. Appl Environ Microbiol.

[CR196] Tracy BP, Jones SW, Papoutsakis ET (2011). Inactivation of σ^E^ and σ^G^ in *Clostridium acetobutylicum* illuminates their roles in clostridial-cell-form biogenesis, granulose synthesis, solventogenesis, and spore morphogenesis. J Bacteriol.

[CR197] Tracy BP, Jones SW, Fast AG, Indurthi DC, Papoutsakis ET (2012). Clostridia: the importance of their exceptional substrate and metabolite diversity for biofuel and biorefinery applications. Curr Opin Biotechnol.

[CR198] Ugwuodo CJ, Nwagu TN (2020). Stabilizing enzymes by immobilization on bacterial spores: A review of literature. Int J Biol Macromol.

[CR199] Varga J, Stirewalt VL, Melville SB (2004). The CcpA protein is necessary for efficient sporulation and enterotoxin gene (*cpe*) regulation in *Clostridium perfringens*. J Bacteriol.

[CR200] Vasylkivska M, Jureckova K, Branska B, Sedlar K, Kolek J, Provaznik I, Patakova P (2019). Transcriptional analysis of amino acid, metal ion, vitamin and carbohydrate uptake in butanol-producing *Clostridium beijerinckii* NRRL B-598. PLoS One.

[CR201] Vees CA, Neuendorf CS, Pflügl S (2020). Towards continuous industrial bioprocessing with solventogenic and acetogenic clostridia: challenges, progress and perspectives. J Ind Microbiol Biotechnol.

[CR202] Wang P, Feng J, Guo L, Fasina O, Wang Y (2019). Engineering *Clostridium saccharoperbutylacetonicum* for high level Isopropanol-Butanol-Ethanol (IBE) production from acetic acid pretreated switchgrass using the CRISPR-Cas9 system. ACS Sustain Chem Eng.

[CR203] Wasels F, Chartier G, Hocq R, Lopes Ferreira N (2020) A CRISPR/Anti-CRISPR genome editing approach underlines the synergy of butanol dehydrogenases in *Clostridium acetobutylicum* DSM 792. Appl Environ Microbiol 86. 10.1128/aem.00408-20 10.1128/AEM.00408-20 PMC7301843 32385078

[CR204] Whitham JM, Tirado-Acevedo O, Chinn MS, Pawlak JJ, Grunden AM (2015). Metabolic response of *Clostridium ljungdahlii* to oxygen exposure. Appl Environ Microbiol.

[CR205] Wilkinson SR, Young M (1994). Targeted integration of genes into the *Clostridium acetobutylicum* chromosome. Microbiology.

[CR206] Wilkinson SR, Young M, Goodacre R, Morris JG, Farrow JAE, Collins MD (1995) Phenotypic and genotypic differences between certain strains of* Clostridium acetobutylicum*. FEMS Microbiol Lett 125(2–3):199–204. 10.1111/j.1574-6968.1995.tb07358.x

[CR207] Williams RHN, Whitworth DE (2010). The genetic organisation of prokaryotic two-component system signalling pathways. BMC Genomics.

[CR208] Wolken WAM, Tramper J, van der Werf MJ (2003). What can spores do for us?. Trends Biotechnol.

[CR209] Woo JE, Lee SY, Jang YS (2018). Effects of nutritional enrichment on acid production from degenerated (non-solventogenic) *Clostridium acetobutylicum* strain M5. Appl Biol Chem.

[CR210] Woods DR, Jones DT (1987). Physiological responses of *Bacteroides* and *Clostridium* strains to environmental stress factors. Adv Microb Physiol.

[CR211] Xin F, Wang C, Dong W, Zhang W, Wu H, Ma J, Jiang M (2016). Comprehensive investigations of biobutanol production by a non-acetone and 1,3-propanediol generating *Clostridium* strain from glycerol and polysaccharides. Biotechnol Biofuels.

[CR212] Xin F, Yan W, Zhou J, Wu H, Dong W, Ma J, Zhang W, Jiang M (2018). Exploitation of novel wild type solventogenic strains for butanol production. Biotechnol Biofuels.

[CR213] Xin X, Cheng C, Du G, Chen L, Xue C (2020). Metabolic engineering of histidine kinases in *Clostridium beijerinckii* for enhanced butanol production. Front Bioeng Biotechnol.

[CR214] Xu M, Zhao J, Yu L, Tang I-C, Xue C, Yang S-T (2015). Engineering *Clostridium acetobutylicum* with a histidine kinase knockout for enhanced n-butanol tolerance and production. Appl Microbiol Biotechnol.

[CR215] Xu M, Zhao J, Yu L, Yang S-T (2017). Comparative genomic analysis of *Clostridium acetobutylicum* for understanding the mutations contributing to enhanced butanol tolerance and production. J Biotechnol.

[CR216] Xu J-Y, Xu Z, Liu X, Tan M, Ye B-C (2018). Protein acetylation and butyrylation regulate the phenotype and metabolic shifts of the endospore-forming *Clostridium acetobutylicum*. Mol Cell Proteomics.

[CR217] Xue Q, Yang Y, Chen J, Chen L, Yang S, Jiang W, Gu Y (2016). Roles of three AbrBs in regulating two-phase *Clostridium acetobutylicum* fermentation. Appl Microbiol Biotechnol.

[CR218] Yang X, Tu M, Xie R, Adhikari S, Tong Z (2013). A comparison of three pH control methods for revealing effects of undissociated butyric acid on specific butanol production rate in batch fermentation of *Clostridium acetobutylicum*. AMB Express.

[CR219] Yang Y, Lang N, Zhang L, Wu H, Jiang W, Gu Y (2020). A novel regulatory pathway consisting of a two-component system and an ABC-type transporter contributes to butanol tolerance in *Clostridium acetobutylicum*. Appl Microbiol Biotechnol.

[CR220] Yu HY, Meade A, Liu SJ (2019). Phylogeny of *Clostridium* spp. Based on conservative genes and comparisons with other trees. Microbiology.

[CR221] Yutin N, Galperin MY (2013). A genomic update on clostridial phylogeny: Gram-negative spore formers and other misplaced clostridia. Environ Microbiol.

[CR222] Zahn JA, Saxena J (2011) Novel ethanologenic *Clostridium* species, *Clostridium coskatii.* In Patent. http://www.google.com/patents/US20110229947

[CR223] Zhang Y, Jiao S, Lv J, Du R, Yan X, Wan C, Zhang R, Han B (2017). Sigma factor regulated cellular response in a non-solvent producing *Clostridium beijerinckii* degenerated strain: a comparative transcriptome analysis. Front Microbiol.

[CR224] Zhang X, Al-Dossary A, Hussain M, Setlow P, Li J (2020). Applications of *Bacillus subtilis* spores in biotechnology and advanced materials. Appl Environ Microbiol.

[CR225] Zhao H, Msadek T, Zapf J, Madhusudan HJA, Varughese KI (2002). DNA complexed structure of the key transcription factor initiating development in sporulating bacteria. Structure.

[CR226] Zhao Y, Tomas CA, Rudolph FB, Papoutsakis ET, Bennett GN (2005). Intracellular butyryl phosphate and acetyl phosphate concentrations in *Clostridium acetobutylicum* and their implications for solvent formation. Appl Environ Microbiol.

[CR227] Zhao R, Liu Y, Zhang H, Chai C, Wang J, Jiang W, Gu Y (2019). CRISPR-Cas12a-mediated gene deletion and regulation in *Clostridium ljungdahlii* and its application in carbon flux redirection in synthesis gas fermentation. ACS Synth Biol.

[CR228] Zheng Y-N, Li L-Z, Xian M, Ma Y-J, Yang J-M, Xu X, He D-Z (2009). Problems with the microbial production of butanol. J Ind Microbiol Biotechnol.

